# Mechanisms of length-dependent recognition of viral double-stranded RNA by RIG-I

**DOI:** 10.1038/s41598-023-33208-w

**Published:** 2023-04-18

**Authors:** Jung Hyun Im, Ivana Duic, Shige H. Yoshimura, Koji Onomoto, Mitsutoshi Yoneyama, Hiroki Kato, Takashi Fujita

**Affiliations:** 1 Division of Integrated Life Science, Graduate School of Biostudies, Kyoto University, Kyoto, 606-8501 Japan; 2 Laboratory of Regulatory Information, Institute for Frontier Life and Medical Sciences, Kyoto University, Kyoto, 606-8397 Japan; 3 Division of Molecular Immunology, Medical Mycology Research Center, Chiba University, Chiba, 260-8673 Japan; 4 Research Institute of Disaster Medicine, Chiba University, Chiba, 260-0856 Japan; 5 Institute for Cardiovascular Immunology, University Hospital Bonn, Bonn, 53127 Germany; 6 R&D Department, xFOREST Therapeutics Co., Ltd., Kyoto, 602-0841 Japan

**Keywords:** Biochemistry, Immunology, Molecular biology

## Abstract

Retinoic acid-inducible gene I (RIG-I) is the most front-line cytoplasmic viral RNA sensor and induces antiviral immune responses. RIG-I recognizes short double-stranded (dsRNA) (< 500 bp), but not long dsRNA (> 500 bp) to trigger antiviral signaling. Since RIG-I is capable of binding with dsRNA irrespective of size, length-dependent RIG-I signaling remains elusive. Here, we demonstrated that RIG-I bound to long dsRNA with slow kinetics. Remarkably, RIG-I/short dsRNA complex efficiently dissociated in an ATP hydrolysis-dependent manner, whereas RIG-I/long dsRNA was stable and did not dissociate. Our study suggests that the dissociation of RIG-I from RIG-I/dsRNA complex could be a step for efficient antiviral signaling. Dissociated RIG-I exhibited homo-oligomerization, acquiring ability to physically associate with MAVS, and biological activity upon introduction into living cells. We herein discuss common and unique mechanisms of viral dsRNA recognition by RIG-I and MDA5.

## Introduction

RIG-I like receptors (RLRs), consist of three members: retinoic acid-inducible gene I (RIG-I), melanoma differentiation-associated protein 5 (MDA5), and laboratory of genetics and physiology 2 (LGP2), are known to function as cytoplasmic viral RNA sensors^1–3^. All three proteins detect and bind to viral RNA using C-terminal domain (CTD) and DExD/H box helicase domain. RIG-I and MDA5 differentially recognize viral RNA and induce valid defensive immune signals by releasing oligomerized N-terminal caspase activation and recruitment domain (CARD). Upon activation, CARD of RIG-I or MDA5 causes CARD-CARD interaction with another CARD-containing mitochondrial antiviral signaling protein (MAVS) to trigger the induction of type I interferon (IFN)^4,5^. On the other hand, LGP2 has been identified as a protein that assists signaling by RIG-I or MDA5 rather than promoting antiviral signaling by itself^1,6^.

RIG-I and MDA5 are activated by different types of RNA. Short double-stranded RNA (dsRNA) (< 500 bp) has been regarded as major RIG-I-stimulatory ligand. Although RIG-I binds to dsRNA regardless of its size, RIG-I is not able to induce antiviral responses with long dsRNA (> 500 bp)^7,8^. On the other hand, MDA5 efficiently senses long dsRNA, but not short dsRNA. MDA5 polymerizes along the length of long dsRNA to form fiber-like polymers^9^. Therefore, it is hypothesized that the formation of long fiber is the critical step for molding MDA5 into the active conformation.

In this study, we aimed to delineate the mechanisms of dsRNA length-dependent RIG-I signaling. We used different length poly (I:C), a co-homopolymer of poly (I) and poly (C) to avoid the influence of sequence specificity. We examined kinetics of the association and dissociation of RIG-I to short and long dsRNA. We found that the dissociation of RIG-I from RIG-I/dsRNA complexes was the key process to transduce signaling, and the dissociation rate was dependent on the length of dsRNA. In fact, we observed that RIG-I rapidly dissociated from short poly (I:C), while RIG-I remained abundantly bound to long poly (I:C) as large aggregates. Also, dissociated RIG-I formed large oligomers, which were capable of associating with MAVS. Furthermore, we demonstrated that dissociated RIG-I induced the dimerization of IRF-3 as well as antiviral gene activation in living cells.

## Results

### Analyses of RIG-I binding with long and short dsRNA

**Figure 1:**
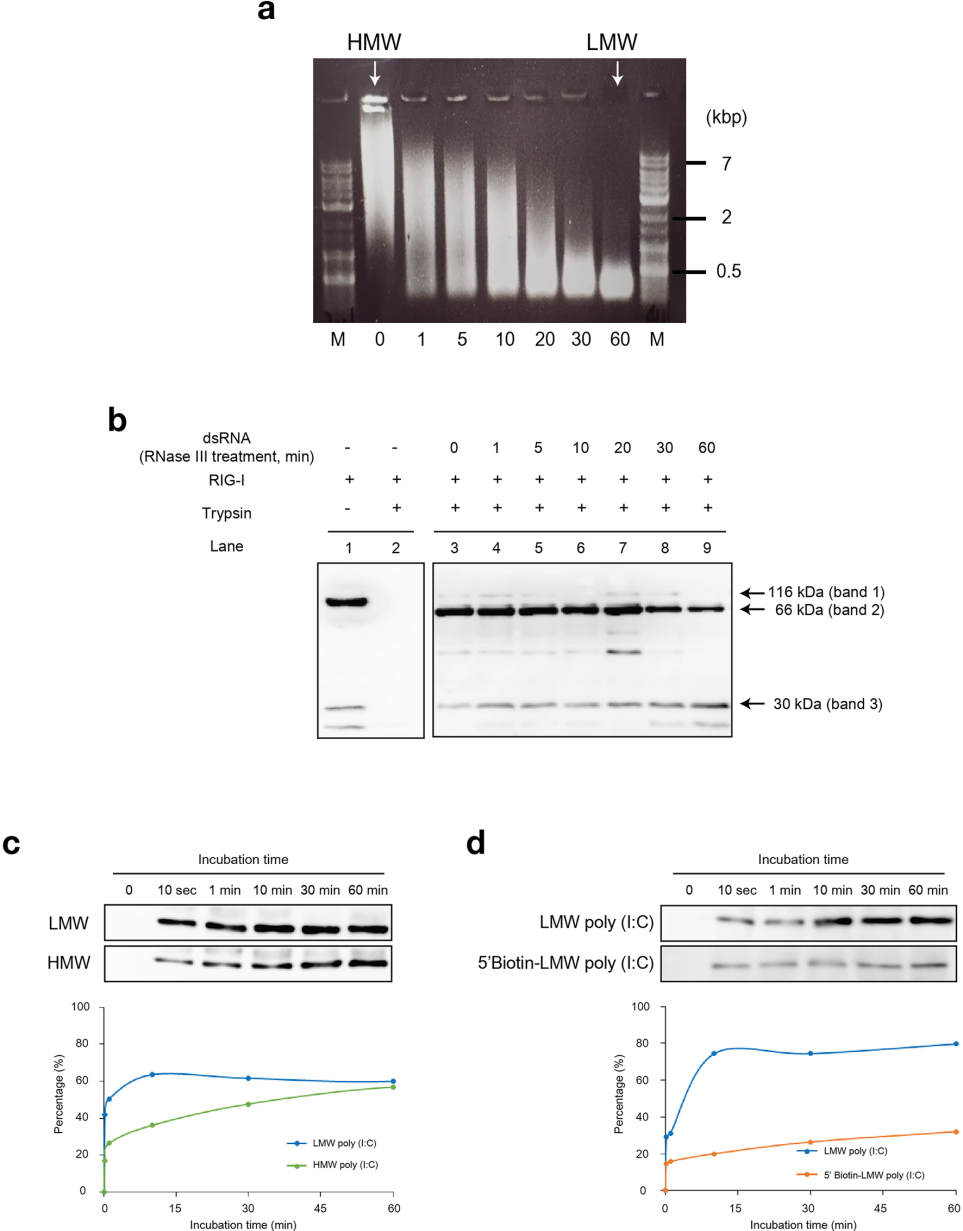
Binding of RIG-I with different lengths of poly (I:C). (**a**) Generation of different sizes of poly (I:C) by RNase III digestion. Commercial poly (I:C) (designated as high molecular weight, HMW) was digested for 0, 1, 5, 10, 20, 30, and 60 min with RNase III to generate different sizes of poly (I:C). The product obtained after 60 min of digestion was used as low molecular weight (LMW) poly (I:C). M: DNA marker. (**b**) Trypsin digestion of RIG-I/poly (I:C) complex. Purified recombinant RIG-I (insect cell) and different sizes of poly (I:C) generated in (**a**) were mixed at 37 °C for 30 min. The reaction mixture was digested with trypsin at 37 °C for 5 min. The reaction mixtures were subjected to SDS-PAGE and immunoblotting with anti-RIG-I (METHODS) as the probe. Undigested RIG-I and RIG-I digested without incubation with poly (I:C) are shown in lanes 1 and 2, respectively. The uncropped original blots are shown in Supplementary Fig. S7a. (**c**) Binding kinetics between RIG-I and poly (I:C) were analyzed. HMW or LMW poly (I:C) was immobilized on magnetic beads through anti-dsRNA antibody. Beads were incubated with recombinant RIG-I for indicated times. RIG-I bound to poly (I:C) was recovered and analyzed by immunoblotting with anti-Flag (top) and band intensity was quantified (bottom). The uncropped original blot is shown in Supplementary Fig. S7b. (**d**) The effect of end modification of poly (I:C) on association with RIG-I. LMW poly (I:C) and biotinylated LMW poly (I:C) (METHODS, 86.6% biotinylated) were subjected to RIG-I binding assay as in (c). The uncropped original blots are shown in Supplementary Fig. S7c.

To elucidate the mechanisms of the size-dependent dsRNA recognition by RIG-I, different lengths of poly (I:C) were generated (Fig. 1a). We designated undigested poly (I:C) as HMW poly (I:C) and that digested for 60 min as LMW poly (I:C) for further experiments. First, recombinant RIG-I was incubated with different lengths of poly (I:C) and digested with trypsin (Fig. 1b). These different sizes of poly (I:C) showed RIG-I-dependent IFNB induction in MDA5 KD HeLa cells consistent with the result from mouse cell line in previous study (Supplementary Fig. S1a and S1b)^7^. RIG-I alone was highly sensitive to trypsin digestion and degraded into small peptides (< 30 kDa, lane 2). In the presence of HMW poly (I:C), a digestion-resistant product of 66 kDa was generated (lanes 3–9). Incubation with shorter poly (I:C) increasingly generated product of 30 kDa. This fragment is particularly increased in the incubation with LMW poly (I:C) (lane 9). These results suggest that poly (I:C) binding induced conformational change of RIG-I and that RIG-I adopted distinct conformation in the complex with HMW and LMW poly (I:C). Previously it was shown that the 30 kDa fragment corresponds to CTD of RIG-I by sequence analysis^10^. Therefore, suggesting that with long poly (I:C), non-CTD region of RIG-I participates in the interaction. In summary, RIG-I interacted with dsRNA regardless of its length, however, the mode of the interaction may differ depending on the size of dsRNA.

We next investigated the binding kinetics of RIG-I with same mass of LMW and HMW poly (I:C) as described in METHODS (Fig. 1c). LMW poly (I:C) bound to RIG-I rapidly whereas HMW poly (I:C) bound to RIG-I slowly. Since the same mass of HMW and LMW poly (I:C) were used for binding assay, the number of ends was higher with LMW poly (I:C); therefore, we speculated that dsRNA end is critical for binding with RIG-I. To test this hypothesis, the 5’ ends of LMW poly (I:C) were biotinylated and examined for their binding with RIG-I (Fig. 1d). Biotinylated LMW poly (I:C) virtually lost binding to RIG-I as considering the efficiency of biotinylation (86.62%). These results suggested that RIG-I binds to dsRNA through termini of dsRNA, resulting in slow association rates with HMW poly (I:C).

### Analyses of dissociation kinetics of dsRNA/RIG-I complex

**Figure 2:**
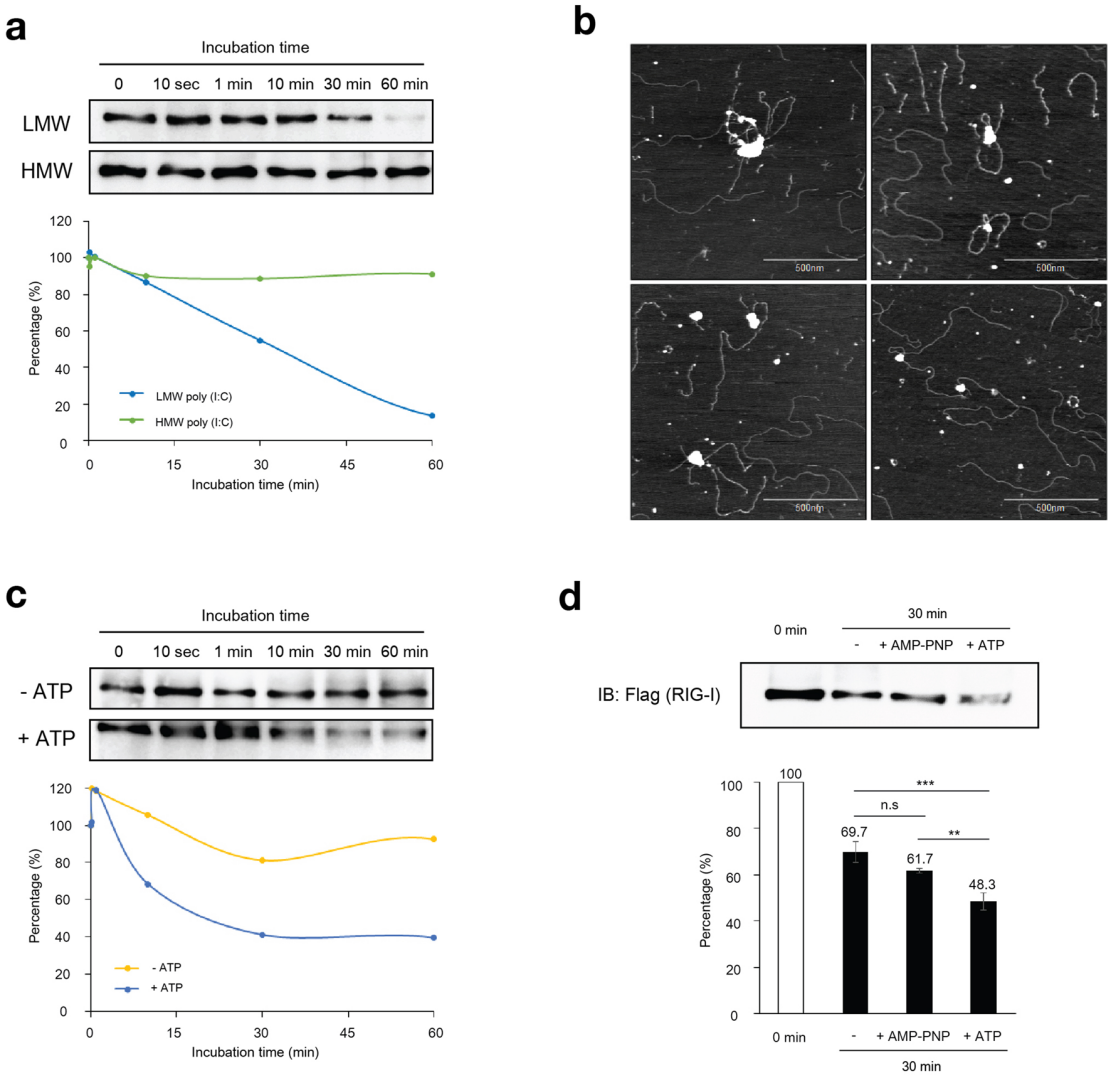
Dissociation of RIG-I/poly (I:C) complex in the presence of ATP. (**a**) Recombinant RIG-I (insect cells) was incubated with LMW or HMW poly (I:C) at 37 °C for 60 min as in Fig. 1C. RIG-I/poly (I:C) complexes on magnetic beads were collected. After washing the beads three times, ATP was added to the beads at final concentration of 1 mM and incubated at 37 °C for different times. RIG-I/dsRNA complexes remaining on the beads were isolated and analyzed by immunoblotting using anti-Flag (top). The dissociation kinetics was determining by quantification of the band intensities (bottom). The uncropped original blots are shown in Supplementary Fig. S8a. (**b**) AFM observation of RIG-I/HMW poly (I:C) complex. RIG-I and HMW poly (I:C) were incubated at 37 °C for 30 min, then further incubated in the presence of 1 mM ATP and subjected to AFM analysis. Four representative fields are shown. For size comparison, see AFM image of naïve RIG-I in Fig. 3A. (**c**) The effect of ATP on the dissociation of RIG-I/LMW poly (I:C) complex was analyzed. The RIG-I/LMW poly (I:C) complexes formed on the magnetic beads were isolated and incubated in the absence or presence of 1 mM ATP at 37 °C for different times. RIG-I bound to poly (I:C) (beads) was analyzed by immunoblotting as in (**a**). The uncropped original blots are shown in Supplementary Fig. S8b. (**d**) The effect of AMP-PNP on dissociation of RIG-I/LMW poly (I:C) complex. RIG-I/LMW poly (I:C) complex was incubated in the absence or presence of 1 mM AMP-PNP or ATP and analyzed as in (**a**). The uncropped original blot is shown in Supplementary Fig. S8c. The percentage of association and dissociation was calculated from band intensities (bottom). Error bars represent standard deviation (n = 3). Data were analyzed using one-way ANOVA followed by Tukey’s post-hoc test (ns, not significant, ** *P* < 0.01 and ***P < 0.001).

We previously reported that dissociation of MDA5 from MDA5/dsRNA complex was critical for adopting active conformation of MDA5 and antiviral signaling^9^. We next examined dissociation kinetics of RIG-I from short and long dsRNA. RIG-I bound with immobilized LMW or HMW poly (I:C) and was washed to remove unbound RIG-I. The complexes were incubated in the presence of 1 mM ATP and RIG-I remaining as complex was quantified (Fig. 2a). The results showed that RIG-I bound to LMW poly (I:C) dissociated rapidly, whereas RIG-I/HMW poly (I:C) complex was stable. AFM observation of the RIG-I/HMW poly (I:C) complex remaining after incubation with 1 mM ATP revealed that RIG-I formed large irregular aggregates on HMW poly (I:C) (Fig. 2b). It is important to note that the aggregates were equivalent to 400–2880 RIG-I monomers. Because ATP is critical for promoting dissociation of MDA5/dsRNA complex^9^, we further examine the effect of ATP in the dissociation of RIG-I from LMW poly (I:C) (Fig. 2c). RIG-I dissociated from LMW poly (I:C) in the absence of ATP, however in the presence of ATP, it was dramatically accelerated. We further investigated the role of ATP in the dissociation. RIG-I/LMW poly (I:C) complex was incubated in the absence or presence of ATP or unhydrolyzable analogue, AMP-PNP (Fig. 2d). Furthermore, ATPase-deficient RIG-I mutant, RIG-I K270A, did not dissociate even in the presence of ATP (Supplementary Fig. S2). Similar to the results shown in Fig. 2c, the presence of 1 mM ATP significantly accelerated the dissociation, however in the presence of AMP-PNP, the dissociation was not significantly augmented. These results indicated that RIG-I bound to LMW poly (I:C) dissociated more efficiently than that bound to HMW poly (I:C) and the dissociation was enhanced by ATP in the hydrolysis-dependent manner.

### Morphological analysis of RIG-I molecule by AFM

**Figure 3:**
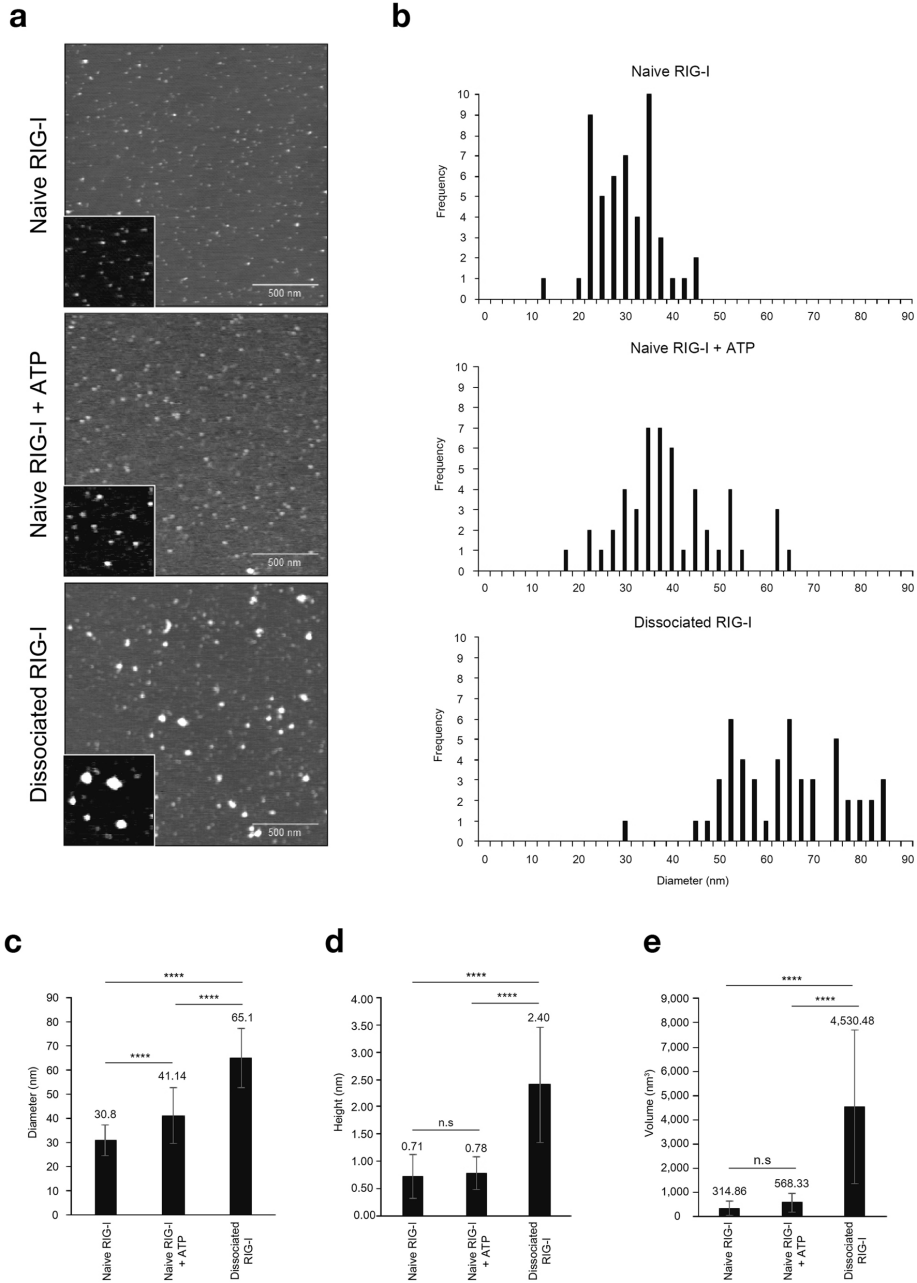
Morphological analysis of RIG-I protein by AFM. (**a**) Purified Flag-tagged RIG-I expressed in 293 T cells (naïve RIG-I, METHODS) was analyzed by AFM (top). Naïve RIG-I was incubated with 1 mM ATP (middle). Naïve RIG-I was bound to immobilized LMW poly (I:C) and incubated with 1 mM ATP for 30 min and released RIG-I (dissociated RIG-I) was recovered and subjected to AFM analysis (bottom). Enlarged images are shown as inset (500 × 500 nm^2^). (**b**) The diameter of objects observed by AFM were measured and plotted as histogram. Average diameter (**c**), height (**d**) and volume (**e**) were similarly determined. Error bars represent ± SD (n = 50). Data were analyzed using one-way ANOVA followed by Tukey’s post-hoc test (ns, not significant, ****P < 0.0001).

Previous analyses of MDA5 demonstrated that after forming fiber-like complexes with long dsRNA, MDA5 was released from dsRNA and adopted distinct conformation in which CARD was exposed, and that the released MDA5 formed multimer^9^. Analogy with this, we hypothesized that RIG-I conforms multimeric complexes upon release from LMW poly (I:C). To validate this, we produced RIG-I in human 293 T cells and purified (METHODS). The recombinant protein (designated as naïve RIG-I) was bound to LMW poly (I:C) in vitro and dissociated RIG-I was recovered (designated dissociated RIG-I, METHODS). Naïve and dissociated RIG-I molecules were subjected to AFM analyses (Fig. 3). Because dissociated RIG-I preparation contained 1 mM ATP, naïve RIG-I in the presence of 1 mM ATP was analyzed for comparison. Under AFM, naïve MDA5 appeared to be composed of 3 structural domains connected with linkers^9^. Unlike MDA5, naïve RIG-I appeared as a single globular shape (diameter of 30.8 nm) (Fig. 3a). In the presence of 1 mM ATP, the diameter marginally increased (41.14 nm). In contrast, dissociated RIG-I appeared as larger bodies (diameter of 65.1 nm). In addition to diameter, the height and volume of dissociated RIG-I were significantly increased. These results suggested that dissociated RIG-I formed multimers.

### Biochemical characterization of dissociated RIG-I

**Figure 4:**
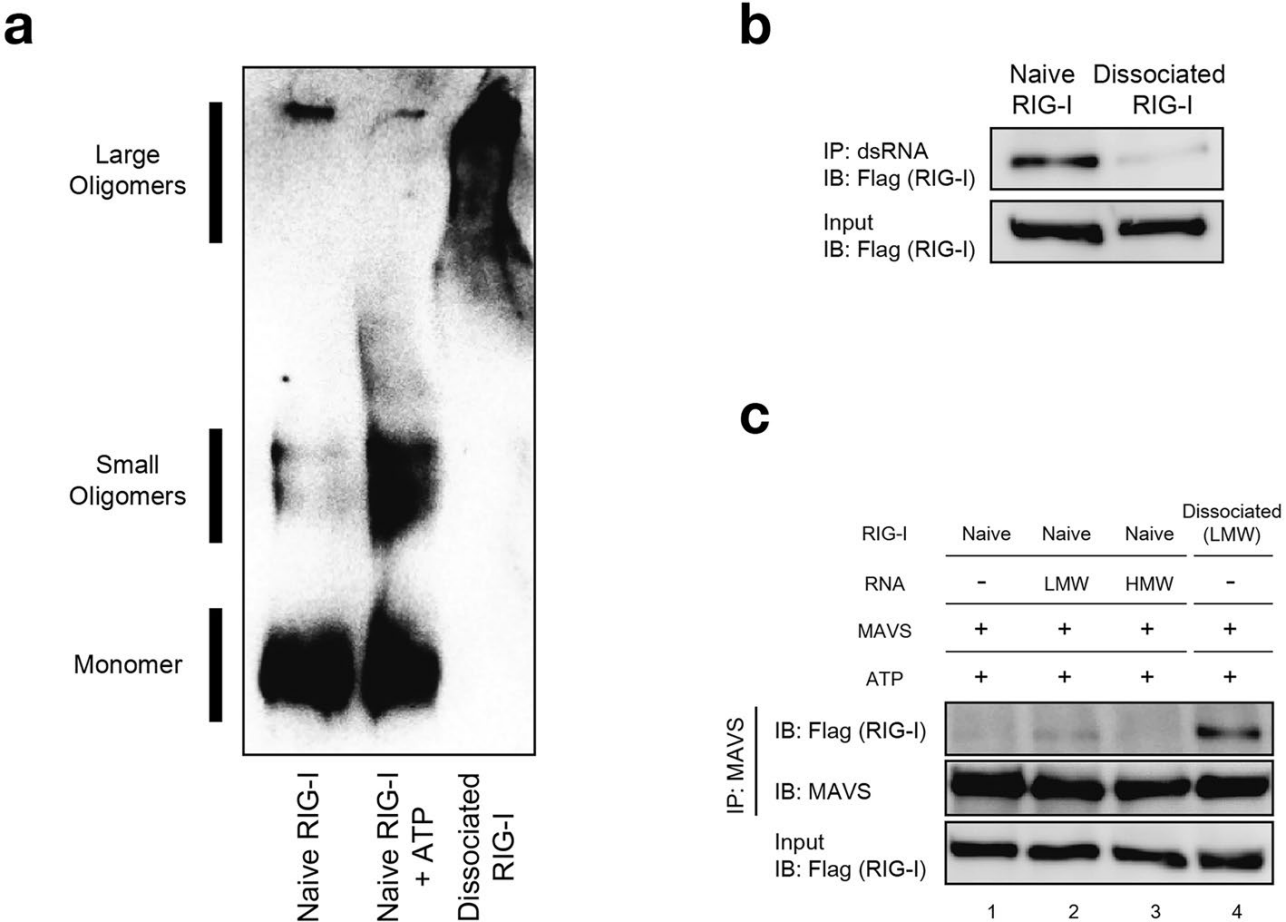
Biochemical characterization of dissociated RIG-I. (**a**) Native PAGE analysis of naïve RIG-I and dissociated RIG-I (293 T cells). Naïve RIG-I in the absence or presence of 1 mM ATP and dissociated RIG-I were applied to native PAGE and analyzed by immunoblotting by anti-Flag. The positions of monomers, small oligomers and large oligomers are indicated. The uncropped original blot is shown in Supplementary Fig. S9a. (**b**) Binding activity of naïve or dissociated RIG-I to LMW poly (I:C) was examined. Recombinant naïve and dissociated RIG-I (20 ng each) were subjected to binding assay with LMW poly (I:C). After incubation at 37 °C for 30 min, RIG-I associated with magnetic beads was analyzed by immunoblotting (IP: dsRNA; IB: Flag (RIG-I)). Input: total amount of naïve and dissociated RIG-I subjected to the binding assay. The uncropped original blots are shown in Supplementary Fig. S9b. (**c**) Physical interaction of RIG-I with MAVS. Naïve RIG-I (lanes 1, 2, and 3) or RIG-I dissociated from LMW poly (I:C) (lane 4) was mixed with recombinant MAVS (***Δ***TM, METHODS). LMW and HMW poly (I:C) were included in lanes 2 and 3, respectively. After incubation at 37 °C for 30 min, the mixtures were subjected to immunoprecipitation with anti-MAVS, followed by immunoblotting with anti-Flag (RIG-I) and anti-MAVS. Input: total amount of RIG-I subjected to the assay. The uncropped original blots are shown in Supplementary Fig. S9c.

Next, we biochemically characterized dissociated RIG-I. First, RIG-I preparations were subjected to native gel electrophoresis (Fig. 4a). Naïve RIG-I was mostly separated as monomeric form with small oligomers as minor species. In the presence of 1 mM ATP, the percentage of small oligomers increased consistent with AFM observation (Fig. 3). The electrophoretic pattern changed dramatically for dissociated RIG-I. Dissociated RIG-I was virtually devoid of monomeric and small oligomers and detected as larger oligomers. We examined if dissociated RIG-I retained binding activity to LMW poly (I:C) (Fig. 4b). Unlike naïve RIG-I, dissociated RIG-I exhibited little binding activity to LMW poly (I:C). We next examined physical association between RIG-I and the mitochondrial adaptor MAVS, which associate through heteromeric CARD-CARD interaction (Fig. 4c). Naïve RIG-I and MAVS were incubated in the absence or presence of LMW or HMW poly (I:C). The interaction between naïve RIG-I and MAVS was undetectable, suggesting that CARD was masked in naïve RIG-I. Similarly, RIG-I complexed with LMW or HMW poly (I:C) showed very weak or no physical interaction with MAVS respectively since enough amounts of dissociated RIG-I cannot be produced in limited time. In contrast, dissociated RIG-I exhibited clear interaction with MAVS, suggesting that CARD was unmasked in dissociated RIG-I. These results strongly suggested that dissociated RIG-I is the signaling competent, active form.

### Biological activity of dissociated RIG-I

**Figure 5:**
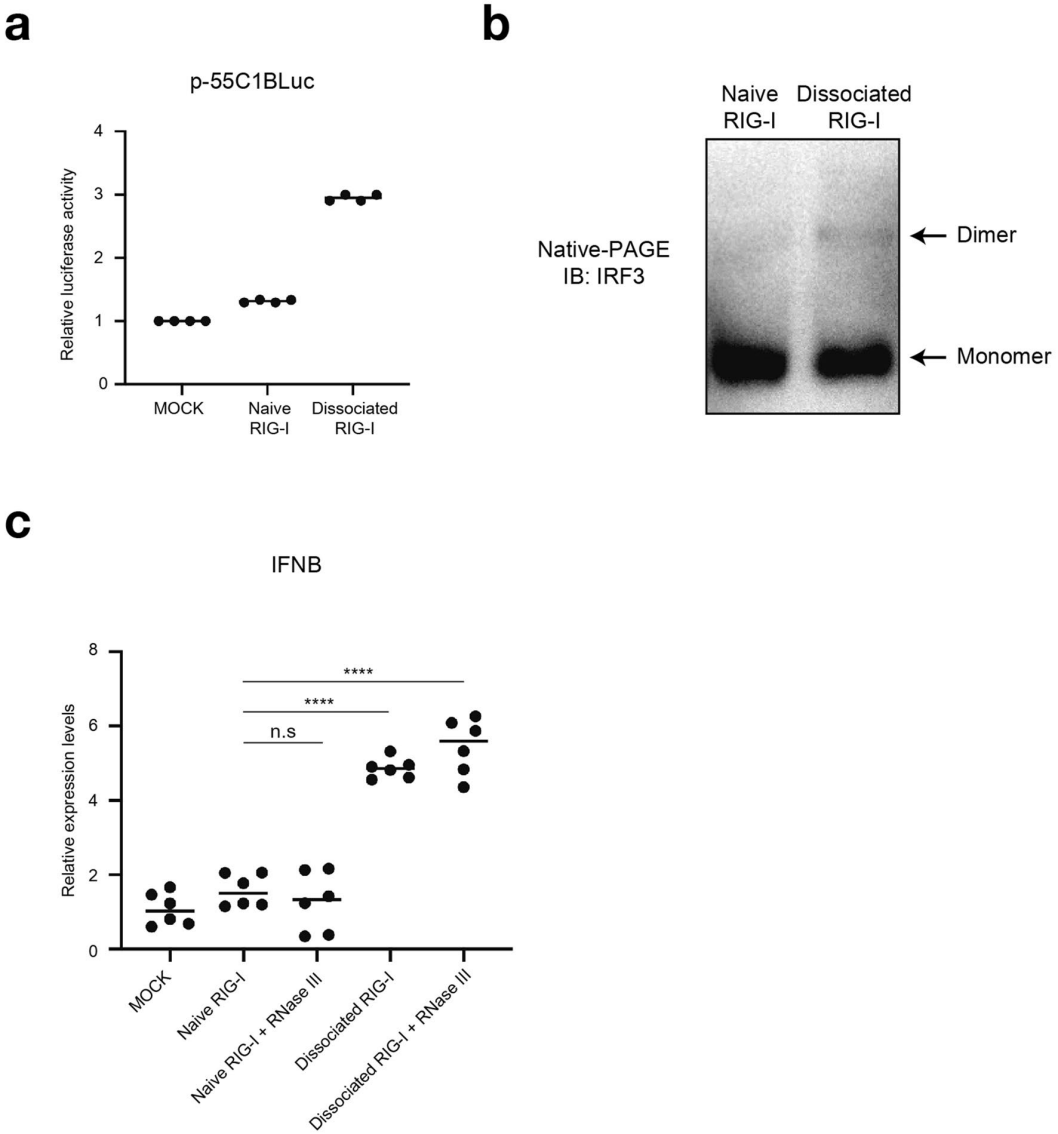
Biological activity of dissociated RIG-I in cells. (**a**) 293 T cells were transfected with p-55C1BLuc, reporter gene containing repetitive IRF3 binding sites, and pRL-TK as an internal control vector. Twenty-four hours after transfection, cells were transfected with 1 µg of naïve or dissociated RIG-I protein, isolated from MDA5 KO 293 T cells, by protein transfection reagent for 2 h (METHODS). After 4 h cultivation with fresh medium, cell extracts were prepared for dual-luciferase assay. Relative firefly luciferase activity normalized by Renilla luciferase activity is shown. Individual values from duplicated samples in each of 2 independent experiments (dots) and average (bar) were shown. (**b**) The cell extracts were analyzed by native PAGE for IRF-3 dimer formation. The positions of monomers and dimers are indicated. The uncropped original blot is shown in Supplementary Fig. S10. (**c**) Naïve or dissociated RIG-I protein, isolated from MDA5 KO 293 T cells, was transfected into HeLa by after 30 min incubation with RNase III. The expression level of IFNB was analyzed by qPCR. Individual values from duplicated samples in each of 3 independent experiments (dots) and average (bar) were shown. Data were analyzed using one-way ANOVA followed by Tukey’s post-hoc test (ns, not significant, ****P < 0.0001).

Finally, we addressed biological activity of dissociated RIG-I in cells. We initially attempted to collect dissociated RIG-I in sufficient amount for protein transfection, however we failed to do so due to technical reasons. To overcome this, we transfected LMW poly (I:C) in MDA5 KO 293 T cells overexpressing RIG-I. Cell extract was prepared and co-precipitated with LMW poly (I:C) and RIG-I, and RIG-I was recovered by incubation with ATP (METHODS). The isolated RIG-I was confirmed by SDS-PAGE and silver staining (Supplementary Fig. S3). Native PAGE revealed that RIG-I isolated as above was in large oligomeric forms (Supplementary Fig. S4). To confirm biological ability of dissociated RIG-I in cells, 293 T cells were transfected with a reporter plasmid containing 8 repeats IRF3 binding site^1,11^. Twenty-four hours after transfection, 293 T cells treated with transfection reagent or transfected with naïve RIG-I exhibited basal reporter gene activity (Fig. 5a). When dissociated RIG-I was transfected, significantly elevated reporter gene activity was detected. Consistent with this, IRF-3 dimer, an active form after its phosphorylation was detected in cells introduced with dissociated RIG-I, but not with naïve RIG-I (Fig. 5b). Also, only dissociated RIG-I and RNase III treated dissociated RIG-I to remove possibility of residual dsRNA could induce IFN-β expression (Fig. 5c and Supplementary Fig. S5). These results strongly support the hypothesis that naïve RIG-I undergoes step by step activation, including binding with LMW dsRNA and ATP hydrolysis-dependent dissociation and conformational changes.

## Discussion

**Figure 6:**
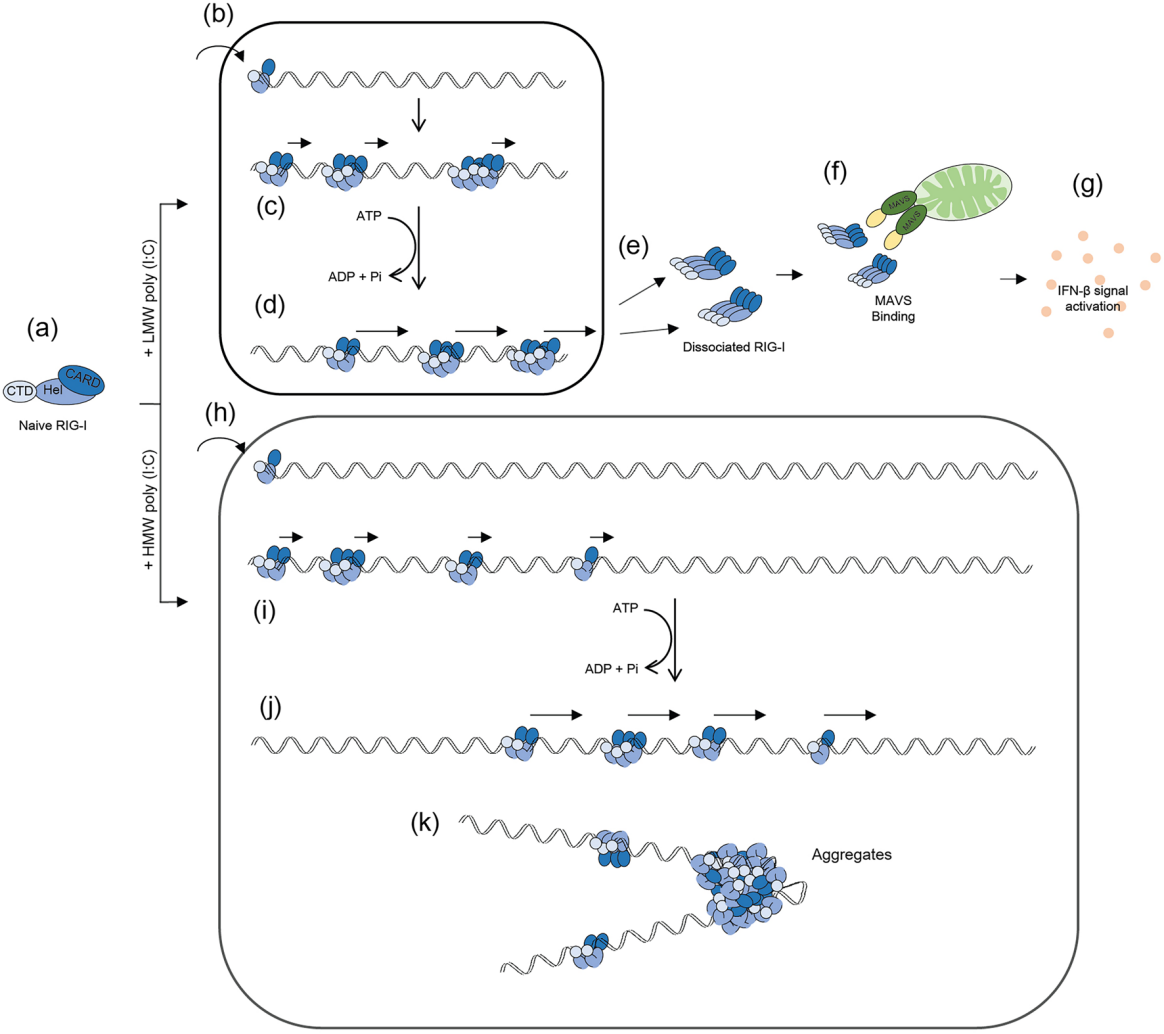
Model for dsRNA length-dependent activation of RIG-I. (**a**) Naïve RIG-I mostly retains closed monomeric conformation where CARD is masked. CARD: caspase activation and recruitment domain, Hel: helicase domain, CTD: C-terminal domain. (**b**) RIG-I recognizes termini of short dsRNA and initiates binding. (**c**) RIG-I translocates along the length of short dsRNA and initiates oligomer formation. (**d**) RIG-I hydrolyzes ATP, accelerating RIG-I translocation on dsRNA and resulting in dissociation from termini. (**e**) The dissociated RIG-I from short dsRNA maintains its oligomeric form. (**f**) Dissociated RIG-I binds to MAVS on mitochondria, resulting in MAVS oligomerization and recruitment of downstream signaling molecules. (**g**) Oligomerization of MAVS induces IFN-β signals by activation of downstream signals, including TBK1 and IRF3. (**h**) RIG-I recognizes termini of long dsRNA and initiates binding with slower kinetics because of limited number of termini. (**i**) RIG-I translocates along the length of long dsRNA. (**j**) RIG-I hydrolyses ATP, accelerating RIG-I translocation on dsRNA. Because of long distance to travel, little RIG-I dissociation takes place. (**k**) In addition, during translocation on long dsRNA, RIG-I forms irregular aggregates that block its translocation and dissociation.

Here, we propose a new model describing how RIG-I recognizes short dsRNA efficiently and is refractory to long dsRNA for triggering antiviral signaling to MAVS. We initially showed that binding kinetics of RIG-I to dsRNA were dependent on its length (Fig. 1). RIG-I bound to LMW dsRNA with fast kinetics, however its binding to HMW dsRNA was slow, but ultimate binding per dsRNA weight was comparable to that of LMW dsRNA. We hypothesize that RIG-I initiates binding from termini of dsRNA. The observation that RIG-I hardly bound to terminally biotinylated dsRNA supports this hypothesis. We also discovered that dissociation kinetics of RIG-I from dsRNA were dependent on its length (Fig. 2). RIG-I/LMW dsRNA dissociated rapidly in the presence of ATP, however RIG-I/HMW dsRNA was stable even in the presence of ATP. It is known that RIG-I scans along the length of dsRNA in an ATP hydrolysis dependent manner^12,13^. Therefore, we propose a model that once RIG-I binds to dsRNA, it moves along dsRNA to encounter another terminus, where to dissociate. Although we do not have confirmatory evidence for this model, our results strongly suggest that dsRNA binding and subsequent dissociation is the critical step for molecular activation of RIG-I. HMW dsRNA is a poor ligand for RIG-I because its binding and dissociation take place inefficiently (Fig. 6a, h-j), as opposed to LMW dsRNA (Fig. 6a-d). Inefficient dissociation is partly because RIG-I entered from termini of dsRNA travels along long dsRNA to dissociate from another end. In addition, we observed irregular aggregates on long dsRNA after incubation with RIG-I (Figs. 2b and 6k). This is in contrast to when long dsRNA was incubated with MDA5, which forms regular fiber-like polymers^9^. These results suggest that during translocation on long dsRNA, RIG-I undergoes irreversible structural changes and become immobile on the RNA.

We next elucidated the nature of “active” RIG-I. Overexpression of CARD in cultured cells is sufficient for induction of type I IFN, but basal activity of full-length RIG-I is undetectable^1^. Therefore, molecular principle of RIG-I activation is unmasking of CARD within RIG-I molecule. In naïve RIG-I, CARD is masked and disruption of “pincer” structure by mutagenesis generated constitutively active RIG-I^14^. Previously, we demonstrated that artificial oligomerization of CARD can mimic RIG-I activation without using dsRNA in living cells^15^. Current study revealed that naïve RIG-I was mostly monomers or small oligomers and RIG-I, which has undergone binding and dissociation with dsRNA, was converted to large oligomers (Fig. 3, 4). Furthermore, dissociated RIG-I exhibited strong association with MAVS, but not naïve RIG-I or RIG-I/LMW dsRNA complex (Fig. 4). Lastly, dissociated RIG-I activated virus-inducible gene promoter and induced IRF-3 dimerization (Fig. 5). Based on these observations, we propose that dissociated RIG-I corresponds to activated RIG-I, which directly transmits signals to MAVS (Fig. 6e-g). This model is consistent with our observation that artificial oligomerization of full-length RIG-I in cells was sufficient to mimic dsRNA-induced signal in an ATP independent manner, suggesting that ATP hydrolysis is dispensable after aggregate formation (Supplementary Fig. S6).

RIG-I and MDA5 sense viral dsRNA with common and unique mechanisms. AFM observation of single MDA5 molecule revealed that MDA5 consists of 3 or 4 structural domains connected with linkers and exhibits dynamic movement in solution^9^. Thus, naïve MDA5 is in extended structure and partially exposes CARD. This is consistent with the observation that MDA5 shows basal activity in over expression in cell but basal activity of RIG-I is undetectable^16^. Unlike RIG-I, MDA5 polymerizes on HMW dsRNA to form fiber-like structure. The polymerization is not restricted to termini of dsRNA, suggesting MDA5 binds to dsRNA independent of its ends. By forming fiber-like polymers, MDA5 changes its conformation into the tighter structure^9^. The fiber dissociation is promoted by the presence of LGP2 in the fiber and ATP hydrolysis^9^. In the case of RIG-I, fiber formation is not a prerequisite for conformational changes and oligomerization. Therefore, RIG-I and MDA5 possess common and distinct mechanisms for recognition of dsRNA and signal activation, thus in combination, capable of sensing variety of viral dsRNA.

## Methods

### Cell culture

293 T (#CRL-3216, ATCC) (female), 293 T MDA5 KO and HeLa cells (#CCL-2.2, ATCC) (female) were maintained in Dulbecco’s modified Eagle’s medium (DMEM) (Nacalai Tesque) supplemented with 10% fetal bovine serum (FBS) and 1% penicillin/streptomycin. 293 T with MDA5 KO was generated by using CRISPR-cas9 system with pSpCas9(BB)-2A-GFP (PX458) backbone plasmid, supported from Dr. Feng Zhang (Addgene plasmid #48,138) as a gift, containing guide RNA (MDA5 sgRNA: forward 5’-TGGTTGGACTCGGGAATTCG-3’ reverse 5’-CGAATTCCCGAGTCCAACCA-3’). Plasmid transfected 293 T was sorted by green fluorescent protein signal using SH800 cell sorter (Sony) and subjected to single-cell selection. Selected cells were cultured as a single clone. L929 cells (#CCL-1, ATCC) were maintained in minimum essential medium (MEM) (Nacalai Tesque) supplemented with 10% FBS and 1% penicillin/streptomycin.

### Poly (I:C)

Poly (I:C) with different lengths were prepared by RNase III (New England Biolabs) digestion of poly (I:C) (HMW, GE Healthcare). HMW poly (I:C) was digested in the reaction mixture (10 µl, 30 µg poly (I:C), 1.5 units RNase III) according to the manufacturer’s protocol at 37 °C. The reaction was stopped by the addition of EDTA. Digested poly (I:C) was recovered by ethanol precipitation.

### Biotinylation of poly (I:C)

LMW poly (I:C) was biotinylated at the 5’-end according to the protocol of 5’ EndTag™ DNA/RNA Labeling Kit (MB-9001, Vectorlabs) using thiol-reactive Biotin Maleimide label (SP-1501, Vectorlabs). The efficiency of biotinylation was calculated by affinity to Streptavidin Mag Sepharose (86.6%).

### Purification of recombinant RIG-I from insect cells

Recombinant RIG-I was produced using baculovirus expression system. High five cells were infected with recombinant baculovirus expressing 6xHis-Flag RIG-I and cultured in SF-900II serum free medium (Invitrogen) supplemented with 1% penicillin/streptomycin. After 72 h infection, RIG-I protein was bound to Ni-Sepharose 6 Fast Flow beads (GE Healthcare) in binding buffer (10 mM imidazole, 50 mM Tris–HCl (pH 8.0), 150 mM NaCl, 5 mM MgCl_2_, and 1.5 mM DTT) and eluted in elution buffer containing 500 mM imidazole. Imidazole was removed using PD-10 desalting column (GE Healthcare).

### Trypsin digestion of RIG-I (insect cells)

A total of 2.7 µg RIG-I in the absence or presence of different lengths of poly (I:C) (2.7 µg) was incubated at 37 °C for 30 min (10 µl) and then treated with 165 ng TPCK-treated trypsin from bovine pancreas (Sigma Aldrich) at 37 °C for 5 min. Trypsin digestion was terminated by adding 2 × SDS sample buffer and boiling for 5 min. The samples were subjected to SDS-PAGE followed by immunoblotting with anti-human RIG-I monoclonal antibody (clone N3514, epitope: aa218-792)^10^. The membranes were cut as proper size prior to immunoblotting with the antibody.

### Binding and dissociation assays for RIG-I-Poly (I:C) complex

Binding assays were performed with binding buffer (20 mM Tris–HCl (pH 8.0), 1.5 mM MgCl_2_, 70 mM KCl and 1.5 mM DTT) at 37 °C for the indicated time. To isolate RIG-I/poly (I:C) complexes, magnetic bead-antibody-dsRNA combination was designed. Protein G magnetic dynabeads (5 µl, Thermo Fisher) were mixed with 1 µg of anti-dsRNA antibody (SCICONS, K1) at room temperature for 40 min. Beads-antibody complexes were washed three times, then 1 µg of poly (I:C) was added and incubated at 4 °C for 1 h. Magnetic bead-antibody-dsRNA complexes were washed three times. Beads were incubated with 1 µg of recombinant RIG-I (insect cells) or purified RIG-I (293 T cells) at 37 °C to generate RIG-I-poly(I:C) complexes for the indicated times. For binding kinetics, the complexes were subjected to SDS-PAGE following immunoblotting with anti-Flag. The membranes were cut as proper size prior to immunoblotting with the antibody. To examine dissociation kinetics, the complexes formed for 60 min were incubated with or without 1 mM ATP for the indicated times and the complexes remained on the beads were analyzed as described above.

### Purification of recombinant RIG-I protein from 293 T cells

293 T cells (1 × 10^6^ cells) were transfected with 5 µg of the expression plasmid pEF-BOS-Flag RIG-I^1^ or pEF-Flag RIG-I K270A^1^ by using Polyethylenimine Max (Polysciences). At 16 h after transfection, the cell extract was prepared with lysis buffer (20 mM Tris–HCl (pH 7.5), 150 mM NaCl, and 1% NP-40 supplemented with protease inhibitors cocktail). The cell lysate was incubated with anti-Flag antibody immobilized to magnetic beads at 4 °C for 16 h. Beads were washed 3 times with lysis buffer and RIG-I was eluted by incubation with of 3 × Flag peptide at room temperature for 30 min. The supernatant was used as naïve RIG-I or RIG-I K270A (293 T).

To isolate dissociated RIG-I from cells, MDA5 KO 293 T cells were transfected with 10 µg of pEF-BOS- Flag RIG-I using Polyethylenimine Max. After 16 h of transfection, 5 µg of LMW poly (I:C) was transfected into the cells for 3 h. Cell lysates were prepared and incubated with magnetic beads coupled with anti-dsRNA antibody at 4 °C for 16 h. Beads were washed 3 times with lysis buffer and mixed with 1 mM ATP at 37 °C for 30 min. Dissociated RIG-I was recovered from the supernatant.

### MAVS ΔTM

MAVS ***Δ***TM was produced in Escherichia coli and purified as previously described^17^.

### AFM

Recombinant RIG-I and HMW poly (I:C) were mixed in AFM buffer (10 µl, 5 mM HEPES–NaOH pH 7.5, 50 mM NaCl, and 5 mM MgCl_2_) incubated at 37 °C for 30 min, and further incubated with 1 mM ATP at 37 °C for 30 min. Then, RIG-I/HMW poly (I:C) was fixed with 0.05% glutaraldehyde at 37 °C for 15 min (Nacalai Tesque) (Fig. 2b). Naïve RIG-I and dissociated RIG-I (293 T cells) were prepared in ATPase buffer (10 µl, 20 mM Tris–HCl pH 7.5, 1.5 mM MgCl_2_, and 1.5 mM DTT) and incubated at 25 °C for 30 min. To examine the effect of ATP on RIG-I, RIG-I incubated in ATPase buffer was further incubated with 1 mM ATP at 37 °C for 30 min (Fig. 3). These samples were placed on 10 mM spermidine (Nacalai Tesque) treated mica at room temperature for 15 min. The mica was washed with Milli-Q and dried thoroughly by blowing with nitrogen gas.

Multimode AFM Nanoscope III a and J scanner (Bruker, Veeco, Digital Instruments) were used for AFM imaging. Samples on mica were scanned with rectangular silicon cantilevers with 14 µm long tetrahedral tips (OMCL-AC160TS-C3, Olympus). Scanned images were analyzed using the software, NanoScope Analysis (v. 5.31 rl, Digital Instruments)^9^.

### Native-PAGE

Naïve or dissociated RIG-I was analyzed using 3–12% Bis–Tris Native PAGE Gels under the NativePAGE Novex Bis–Tris Gel System. Samples were electrophoresed at 200 V at 4 °C for 100 min and transferred to PVDF membranes at 15 V for 1 h. Proteins on the membranes were fixed with 20 ml of 8% acetic acid. The membranes were rinsed according to the Native-PAGE protocol (Life Technologies). RIG-I was visualized by immunoblotting with anti-Flag antibody (RIG-I). The membranes were cut as proper size prior to immunoblotting with the antibody.

Native PAGE of IRF-3 was described elsewhere^18^.

### Luciferase assay and protein transfection

293 T cells were transfected with reporter genes (0.5 µg of p-55C1BLuc and 25 ng of p-RL-TK)^1,11^. After 16 h, naïve or dissociated RIG-I, isolated from MDA5 KO 293 T cells, was transfected into cells using the Xfect protein transfection kit (Takara) for 4 h. Cells were further cultivated for 4 h in fresh medium. Luciferase activities were determined as previously described^9^.

### Real-Time qPCR

The RNA from cells was extracted by TRIzol, and synthesized to cDNA using ReverTra ACE aPCR RT Master Mix including gDNA remover (TOYOBO). cDNA was mixed with primers (Supplementary Table S1) and Thunderbird SYBR real-time PCR Mix (TOYOBO). Real-Time qPCR was performed and analysed using Step One plus real-time PCR system (Applied Biosystems).

### Artificial oligomerization of RIG-I in cells

For oligomerization of RIG-I in cells, we modified an artificial homodimerization system (ARGENT Kit, ARIAD; currently iDimerize system, Clontech). We used 3 tandem repeats of mutant FK 506 Binding Protein 12 (FK_F36V_), which can be cross-linked by the cell-permeable chemical AP20187. FK_F36V_ harbours an F36V mutation, which impairs binding affinity to the immunosuppressive agent, FK506. AP20187 was specifically designed for binding with FK_F36V_; therefore, it does not influence endogenous FK binding proteins. Thus, this system specifically crosslinks a target protein without unwanted side effects. We made constructs to artificially oligomerize full-length RIG-I in cells (FK-RIG-I). L929 cells (5 × 10^5^ cells) were transfected with p-55C1BLuc (1.25 µg, signal reporter), pRL-TK (10 ng, reference reporter) and the expression vector (1.25 µg) for 12 h. Cells were replated into 96 well dishes for 12 h. Cells were stimulated with AP20187 (100 nM) for 9 h. Reporter activities were determined by the dual luciferase assay kit as described^16^.

### Silver staining

Recombinant naïve RIG-I (insect cells or 293 T cells), dissociated RIG-I isolated from MDA5 KO 293 T cells, and 0.5 µg of dsRNA antibody were prepared in binding buffer (15 µl). Samples were subjected to SDS-PAGE after the addition of 2X SDS sample buffer and boiling for 5 min. The SDS gel was stained using Sil-Best Stain One following the kit’s protocol.

### Supplementary Information

Supplementary Information.

## Data Availability

All datasets analyzed in this study are included in the published article and Supplementary Information. The data used and analyzed during the current study is available from the corresponding author with reasonable request.
