# Author Correction: Mechanisms of length-dependent recognition of viral double-stranded RNA by RIG-I

**DOI:** 10.1038/s41598-023-50644-w

**Published:** 2024-01-08

**Authors:** Jung Hyun Im, Ivana Duic, Shige H. Yoshimura, Koji Onomoto, Mitsutoshi Yoneyama, Hiroki Kato, Takashi Fujita

**Affiliations:** 1 Division of Integrated Life Science, Graduate School of Biostudies, Kyoto University, Kyoto, 606-8501 Japan; 2 Laboratory of Regulatory Information, Institute for Frontier Life and Medical Sciences, Kyoto University, Kyoto, 606-8397 Japan; 3 Division of Molecular Immunology, Medical Mycology Research Center, Chiba University, Chiba, 260-8673 Japan; 4 Research Institute of Disaster Medicine, Chiba University, Chiba, 260-0856 Japan; 5 Institute for Cardiovascular Immunology, University Hospital Bonn, Bonn, 53127 Germany; 6 R&D Department, xFOREST Therapeutics Co., Ltd., Kyoto, 602-0841 Japan

Correction to: *Scientific Reports* 10.1038/s41598-023-33208-w, published online 18 April 2023

The original version of this Article contained errors in Figures. 2, 3 and 5 and the legends where the statistical analyses were incorrect. The original Figures 2, 3 and 5 and accompanying legends appear below.

**Figure 2:**
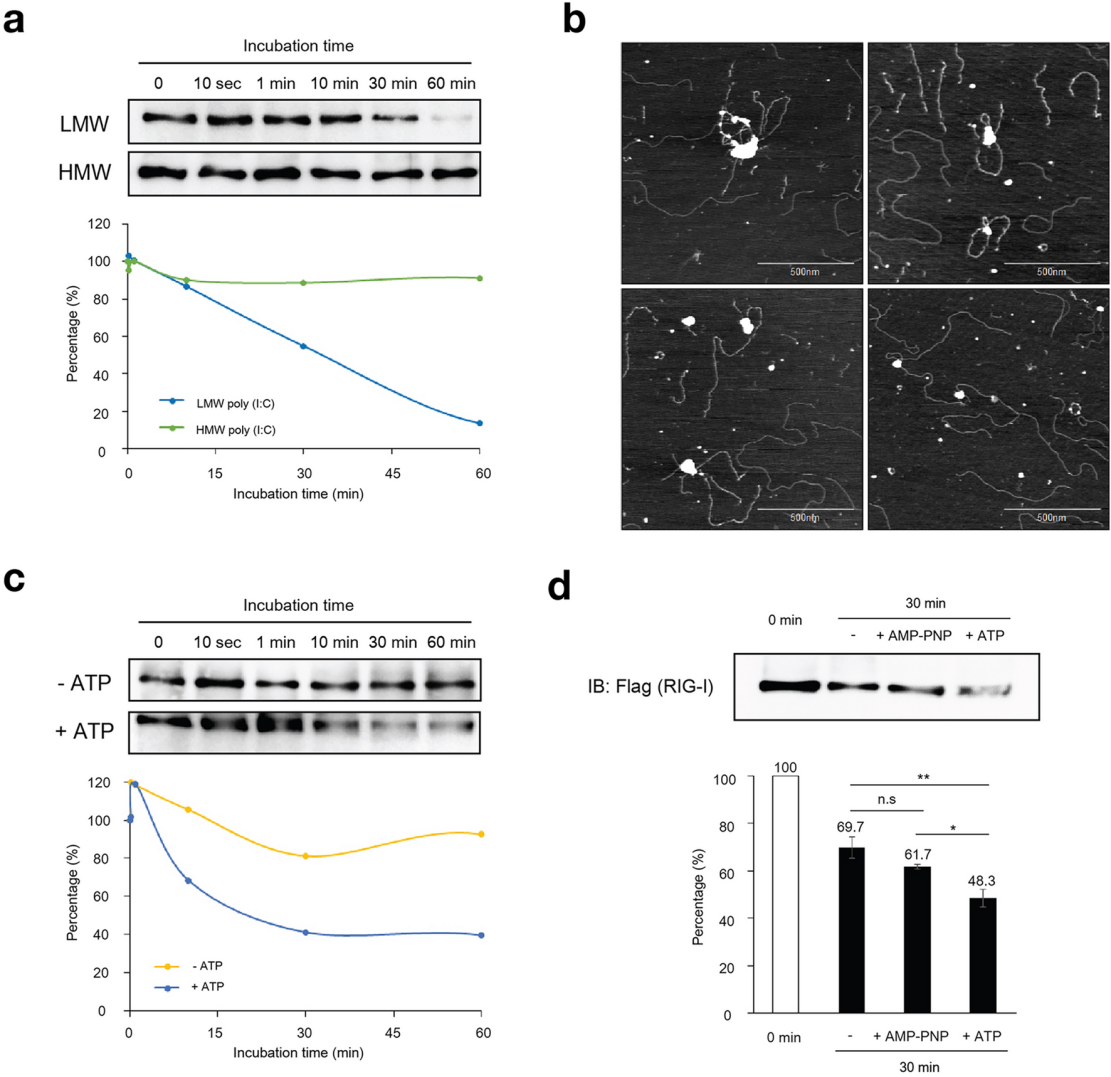
Dissociation of RIG-I/poly (I:C) complex in the presence of ATP. (**a**) Recombinant RIG-I (insect cells) was incubated with LMW or HMW poly (I:C) at 37 °C for 60 min as in Fig. 1C. RIG-I/poly (I:C) complexes on magnetic beads were collected. After washing the beads three times, ATP was added to the beads at final concentration of 1 mM and incubated at 37 °C for different times. RIG-I/dsRNA complexes remaining on the beads were isolated and analyzed by immunoblotting using anti-Flag (top). The dissociation kinetics was determining by quantification of the band intensities (bottom). The uncropped original blots are shown in Supplementary Fig. S8a. (**b**) AFM observation of RIG-I/HMW poly (I:C) complex. RIG-I and HMW poly (I:C) were incubated at 37 °C for 30 min, then further incubated in the presence of 1 mM ATP and subjected to AFM analysis. Four representative fields are shown. For size comparison, see AFM image of naïve RIG-I in Fig. 3A. (**c**) The effect of ATP on the dissociation of RIG-I/LMW poly (I:C) complex was analyzed. The RIG-I/LMW poly (I:C) complexes formed on the magnetic beads were isolated and incubated in the absence or presence of 1 mM ATP at 37 °C for different times. RIG-I bound to poly (I:C) (beads) was analyzed by immunoblotting as in (a). The uncropped original blots are shown in Supplementary Fig. S8b. (**d**) The effect of AMP-PNP on dissociation of RIG-I/LMW poly (I:C) complex. RIG-I/LMW poly (I:C) complex was incubated in the absence or presence of 1 mM AMP-PNP or ATP and analyzed as in (a). The uncropped original blot is shown in Supplementary Fig. S8c. The percentage of association and dissociation was calculated from band intensities (bottom). Error bars represent standard deviation (n = 3). *P < 0.05, . **P < 0.01, unpaired Student’s *t* test. n.s, not significant.

**Figure 3:**
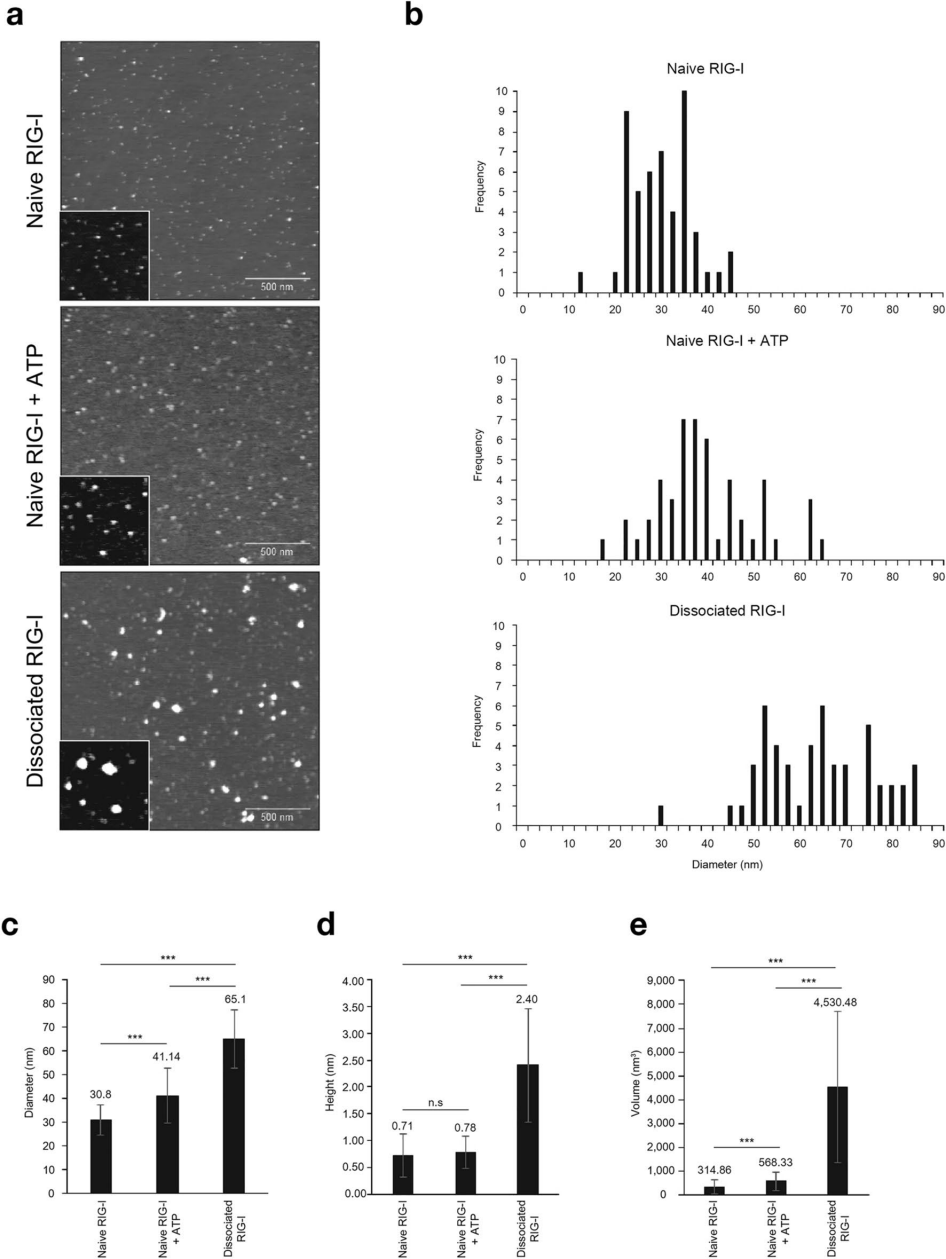
Morphological analysis of RIG-I protein by AFM. (**a**) Purified Flag-tagged RIG-I expressed in 293 T cells (naïve RIG-I, METHODS) was analyzed by AFM (top). Naïve RIG-I was incubated with 1 mM ATP (middle). Naïve RIG-I was bound to immobilized LMW poly (I:C) and incubated with 1 mM ATP for 30 min and released RIG-I (dissociated RIG-I) was recovered and subjected to AFM analysis (bottom). Enlarged images are shown as inset (500 × 500 nm2). (**b**) The diameter of objects observed by AFM were measured and plotted as histogram. Average diameter (**c**), height (**d**) and volume (**e**) were similarly determined. Error bars represent standard deviation (n = 50). ***P < 0.001, unpaired Student’s *t* test. n.s, not significant.

**Figure 5:**
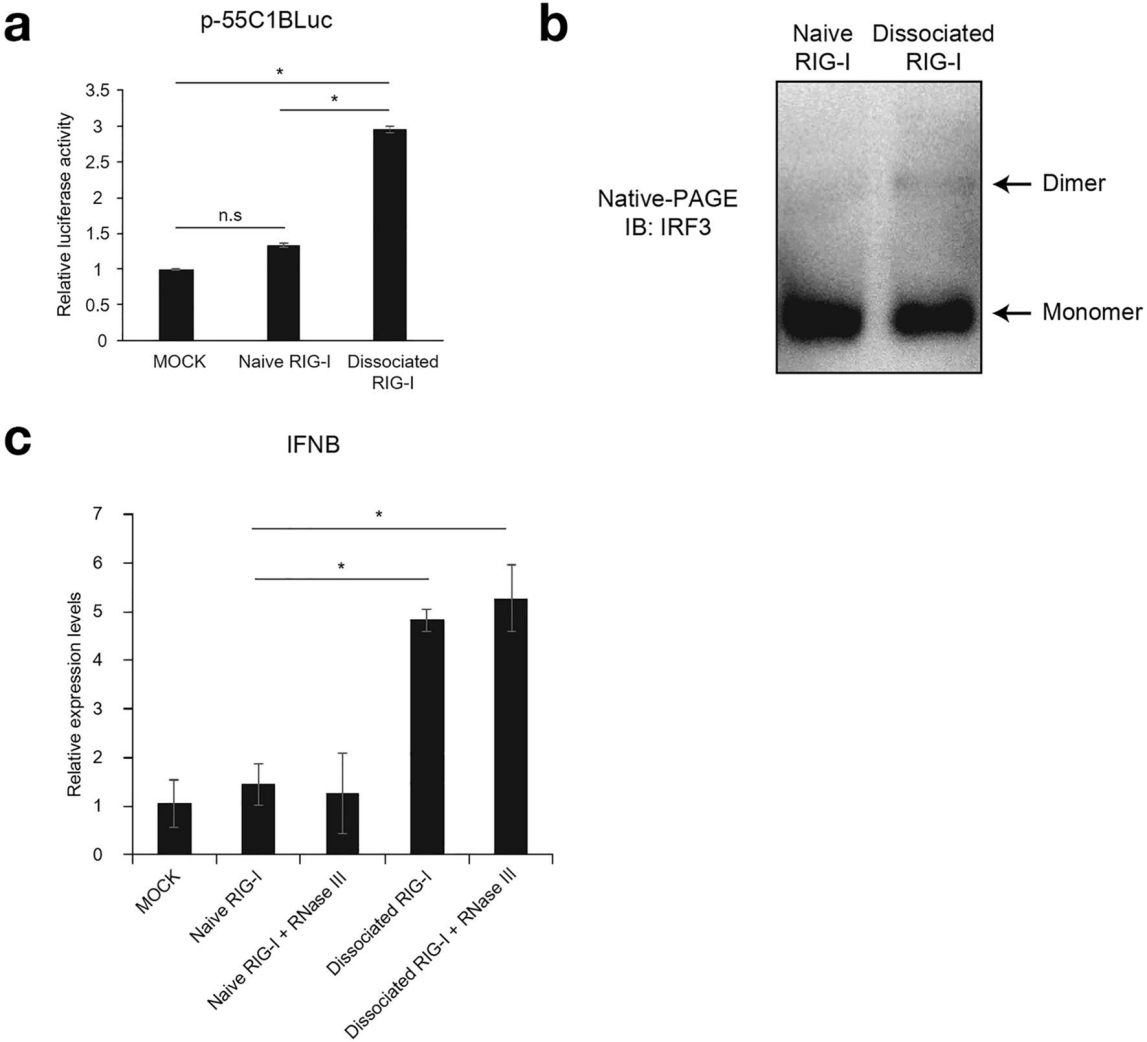
Biological activity of dissociated RIG-I in cells. (**a**) 293 T cells were transfected with p-55C1BLuc, reporter gene containing repetitive IRF3 binding sites, and pRL-TK as an internal control vector. Twenty-four hours after transfection, cells were transfected with 1 µg of naïve or dissociated RIG-I protein, isolated from MDA5 KO 293 T cells, by protein transfection reagent for 2 h (METHODS). After 4 h cultivation with fresh medium, cell extracts were prepared for dual-luciferase assay. Relative firefly luciferase activity normalized by Renilla luciferase activity is shown. Luciferase activity data are the average of 2 independent experiments. Error bars represent standard deviation (n = 2). *P < 0.05, unpaired Student’s t-test. n.s, not significant. (**b**) The cell extracts were analyzed by native PAGE for IRF-3 dimer formation. The positions of monomers and dimers are indicated. The uncropped original blot is shown in Supplementary Fig. S10. (**c**) Naïve or dissociated RIG-I protein, isolated from MDA5 KO 293 T cells, was transfected into HeLa by after 30 min incubation with RNase III. The expression level of IFNB was analyzed by qPCR. Error bars represent standard deviation (n = 2). *P < 0.05, unpaired Student’s t-test.

In addition, the Supplementary Information file published with this Article contained an error in the Supplementary Figure S1. The original Supplementary Information file is provided below.

The original Article and the Supplementary Information file have been corrected.

### Supplementary Information

Supplementary Information.

