# Supplementary material for: Author Correction: Mechanisms of length-dependent recognition of viral double-stranded RNA by RIG-I
Source: Sci Rep. 2024 Jan 8;14:835. doi: 10.1038/s41598-023-50644-w (PMC10774395; doi:10.1038/s41598-023-50644-w)
Supplement: Supplementary file 1 — Supplementary Information. [file 41598_2023_50644_MOESM1_ESM.pdf]

Supplementary Information

Mechanisms of length-dependent recognition of viral double-stranded RNA by RIG-I

Jung Hyun Im[1,2], Ivana Duic[1,2], Shige H. Yoshimura[1], Koji Onomoto[3], Mitsutoshi Yoneyama[3,4], Hiroki Kato[5] and Takashi Fujita[1,2,5,*]

[1]Division of Integrated Life Science, Graduate School of Biostudies, Kyoto University, Kyoto 606-8501, Japan,
[2]Laboratory of Regulatory Information, Institute for Frontier Life and Medical Sciences, Kyoto University, Kyoto 606-8397, Japan,
[3]Division of Molecular Immunology, Medical Mycology Research Center, Chiba University, Chiba 260-8673, Japan,
[4]Research Institute of Disaster Medicine, Chiba University, Chiba 260-0856, Japan,
[5]Institute for Cardiovascular Immunology, University Hospital Bonn, Bonn 53127, Germany

* To whom correspondence should be addressed.
Tel: +81 75 751 4031; Fax: +81 75 751 4031;
Email: fujita.takashi.86e@st.kyoto-u.ac.jp

Supplementary Figure S1

a

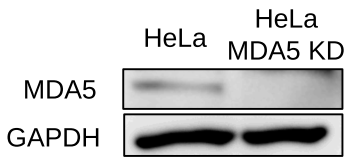

b

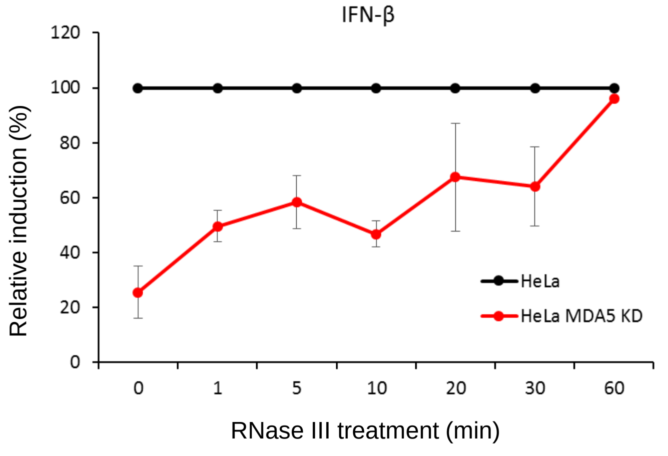

Supplementary Figure S1. (a) Expression of MDA5 was confirmed in WT HeLa and MDA5 knockdown (KD) HeLa. The uncropped gel images are shown in Supplementary Figure S11. (b) Relative induction of IFNB in WT HeLa and HeLa MDA5 KD with transfection of different length of poly (I:C). Different length of poly (I:C) was generated by treating RNase III for indicated time. Error bars represent standard deviation (n=2).

Supplementary Figure S2

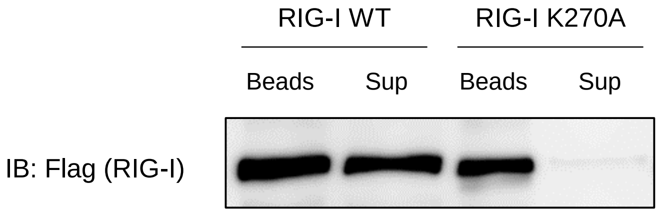

Supplementary Figure S2. Dissociation of RIG-I was induced with wild-type RIG-I and ATP hydrolysis deficient RIG-I mutants, RIG-I K270A. RIG-I was incubated with LMW poly (I:C) at 37° C for 30 min respectively. RIG-I/poly (I:C) complexes were collected and incubated at 37° C for 30 min with ATP. The mixture was fractionated into RIG-I/poly (I:C) (beads) and dissociated RIG-I (supernatant) by pulling down magnetic beads. The fractions were analyzed by anti-Flag antibody (RIG-I).
Sup: Supernatant. The uncropped gel images are shown in Supplementary Figure S11.

Supplementary Figure S3

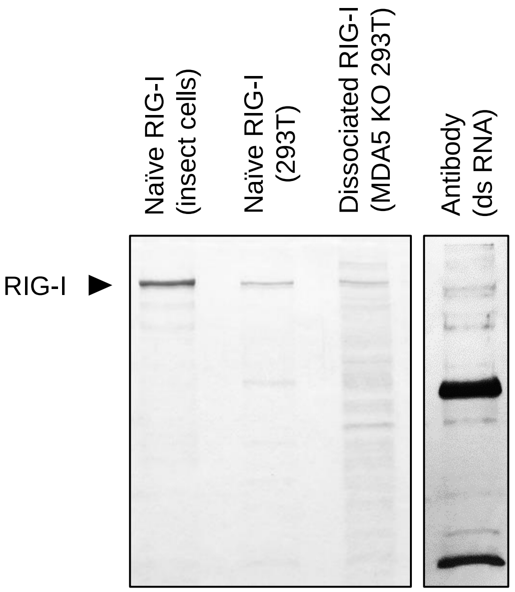

Supplementary Figure S3. Silver staining of naïve RIG-I and MDA5 KO 293T cell-derived dissociated RIG-I. Naïve RIG-I from insect cells, 293T cells, dissociated RIG-I isolated from MDA5 KO 293T cells were subjected to SDS-PAGE and silver staining. The uncropped gel images are shown in Supplementary Figure S11.

Supplementary Figure S4

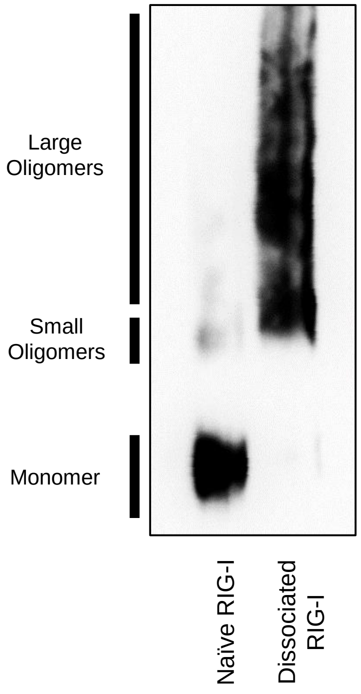

Supplementary Figure S4. Native PAGE analysis of naïve RIG-I and dissociated RIG-I used for protein transfection (Figure 5). Naïve RIG-I (293T cells) and dissociated RIG-I isolated from MDA5 KO 293T cells were subjected to native PAGE and analyzed by immunoblotting by anti-Flag. The positions of monomers, small oligomers and large oligomers are indicated. The uncropped original blot is shown in Supplementary Figure S11.

Supplementary Figure S5

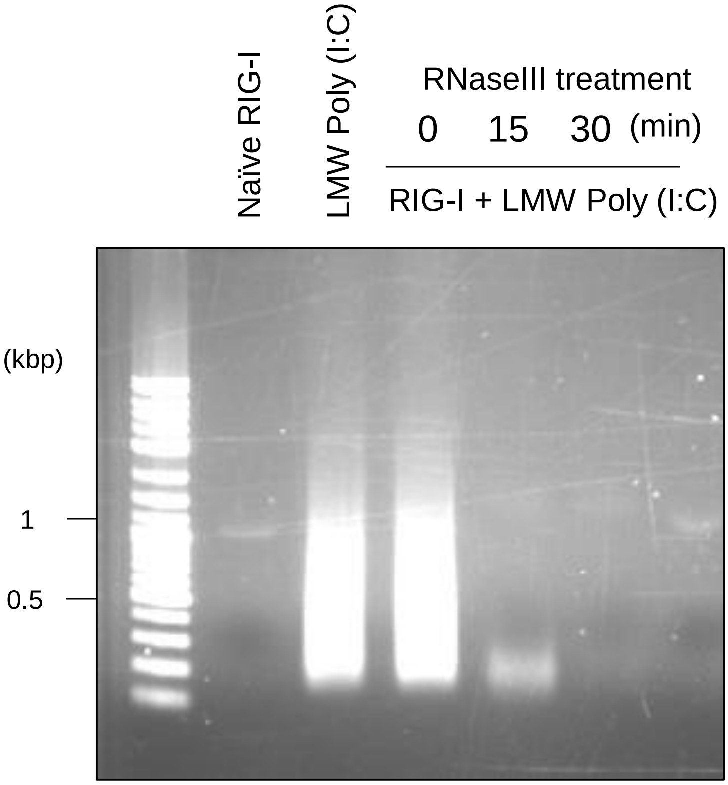

Supplementary Figure S5. Time condition of RNase III treatment to naïve and dissociated RIG-I was optimized. 1 ug of LMW poly (I:C) mixed with RIG-I was fully degraded in 30 min by RNase Ⅲ treatment. The uncropped original blot is shown in Supplementary Figure S11.

Supplementary Figure S6

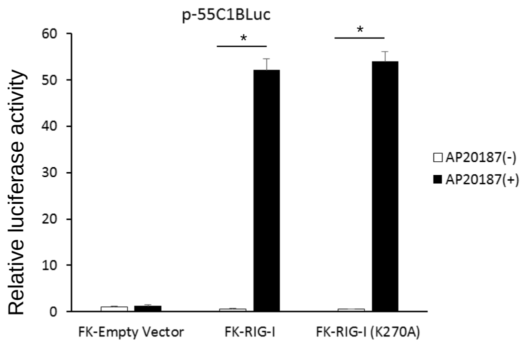

Supplementary Figure S6. Effect of artificial oligomerization on virus-inducible promoter activation. A fusion construct for 3 tandem repeats of FKBP and full-length human RIG-I was prepared. Another fusion construct for FKBP and RIG-I with disrupted ATP binding site (RIG-I K270A) was similarly prepared. L929 cells were transfected with indicated expression vector and reporter genes, p-55C1BLuc and p-RL-TK. Cells were stimulated by the addition of crosslinker AP20187 for 9 h and luciferase activities were measured (METHODS). Data of duplicate assays are presented. Error bars represent standard deviation. * $P < 0.05$, unpaired Student's t-test.

Supplementary Figure S7

a

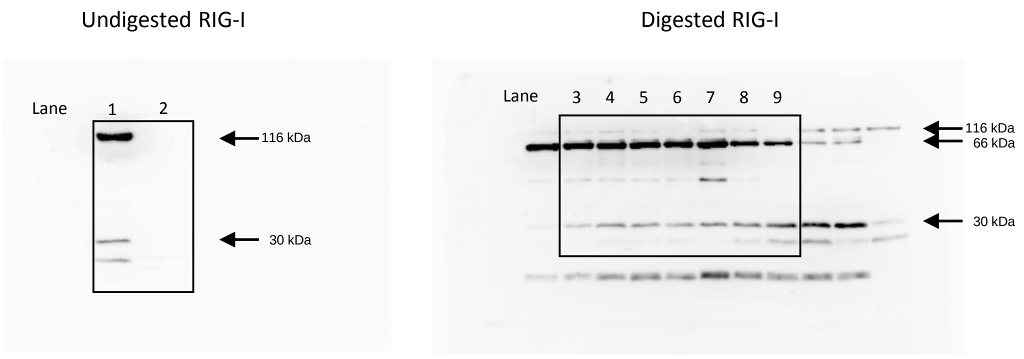

b

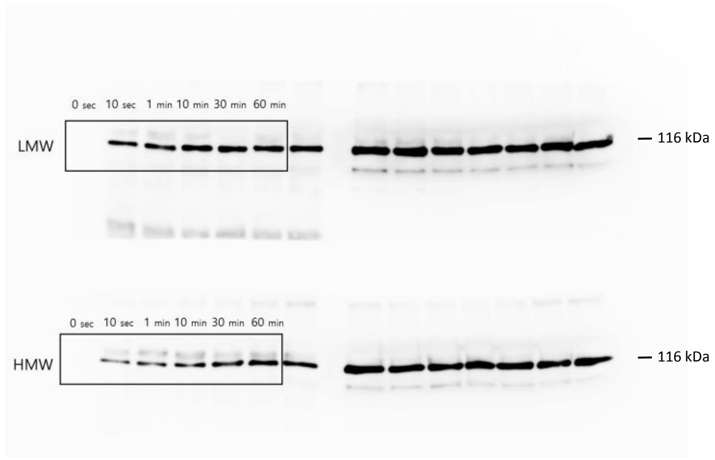

c

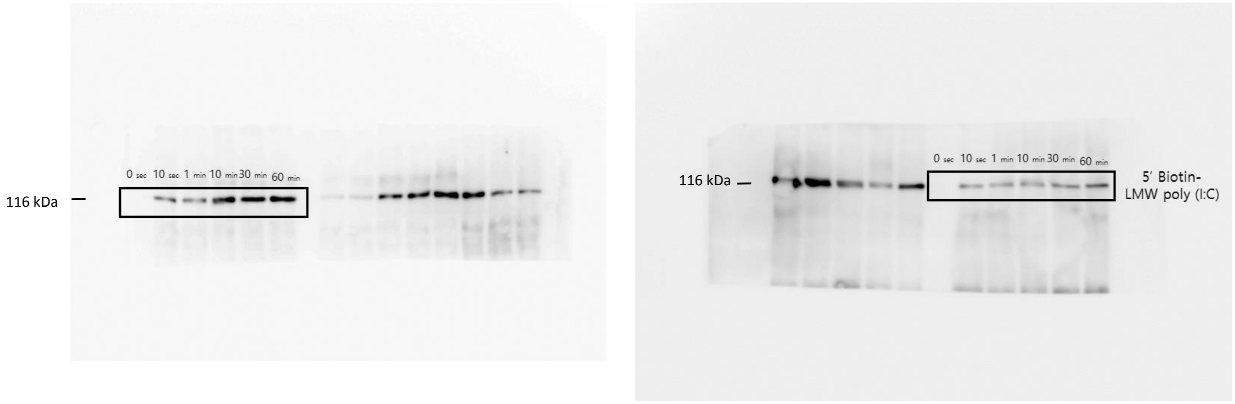

Supplementary Figure S7 (a-c) shows full-size of uncropped SDS-PAGE images in Figure 1b-d respectively. The membranes were cut as proper size prior to immunoblotting with the antibodies. No more additional modification was done.

Supplementary Figure S8

a

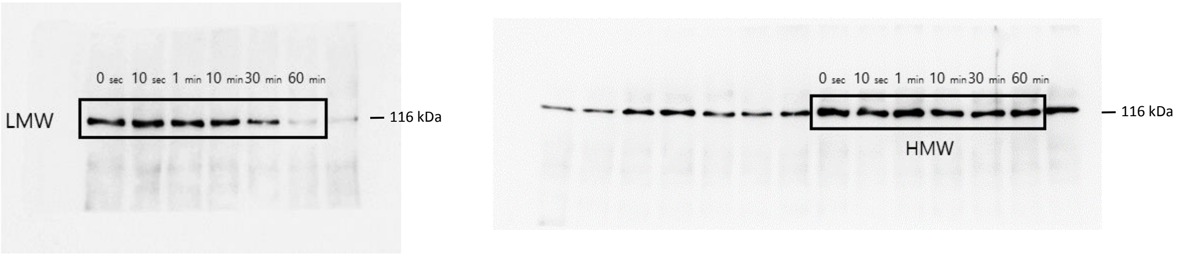

b 1st trial

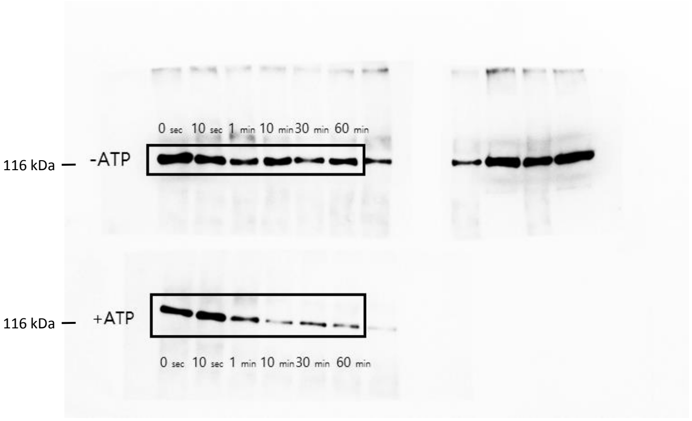

2nd trial

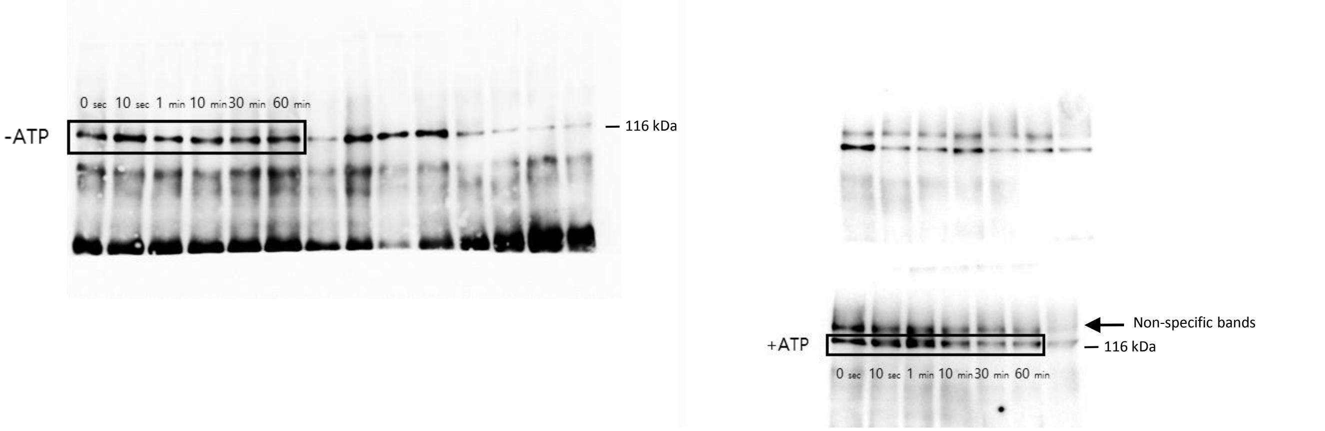

c

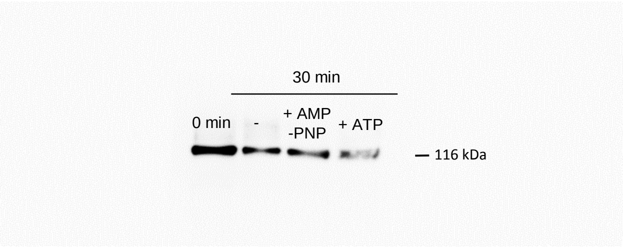

Supplementary Figure S8 (a-c) shows full-size of uncropped SDS-PAGE images in Figure 2 a, c, d respectively. The membranes were cut as proper size prior to immunoblotting with the antibodies. No more additional modification was done.

Supplementary Figure S9

a

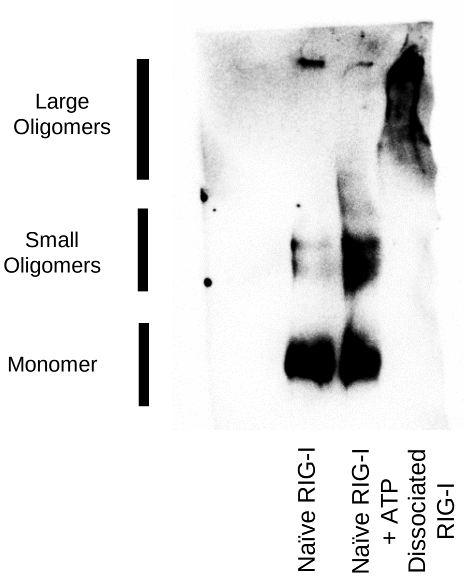

b

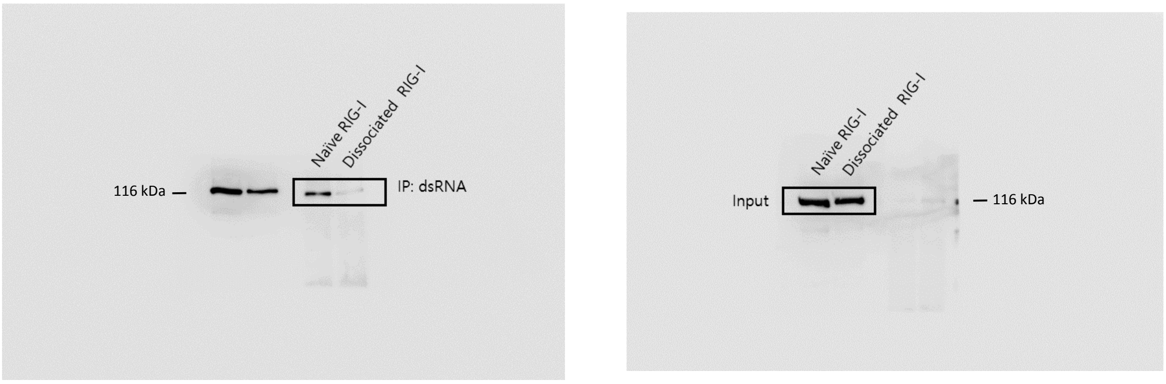

c

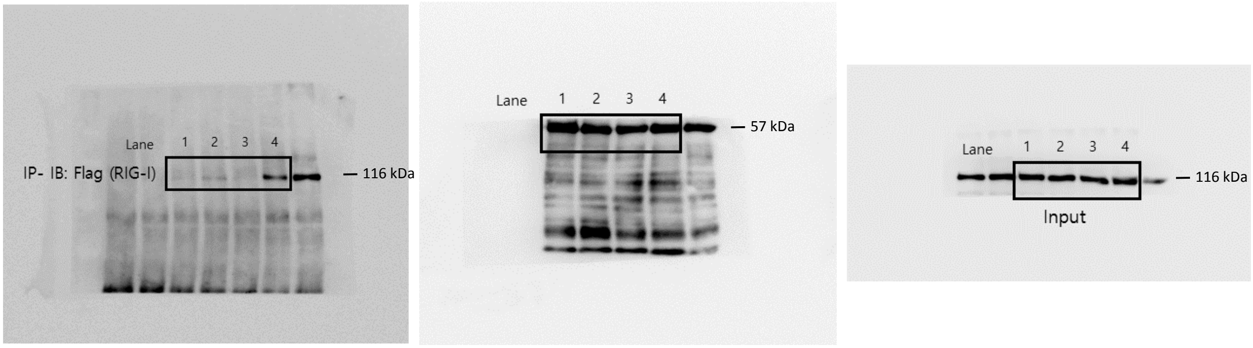

Supplementary Figure S9 (a-c) shows full-size of uncropped Native-PAGE and SDS-PAGE images in Figure 4 a, b and c respectively. The membranes were cut as proper size prior to immunoblotting with the antibodies. No more additional modification was done.

Supplementary Figure S10

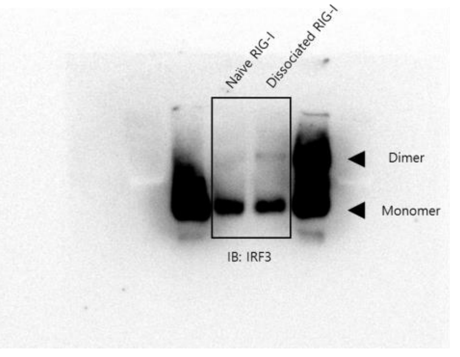

Supplementary Figure S10 shows full size of an uncropped SDS-PAGE image in Figure 5 b. The membrane was cut as proper size prior to immunoblotting with the antibodies. No more additional modification was done.

Supplementary Figure S11

Raw data of Supplementary Fig. S1

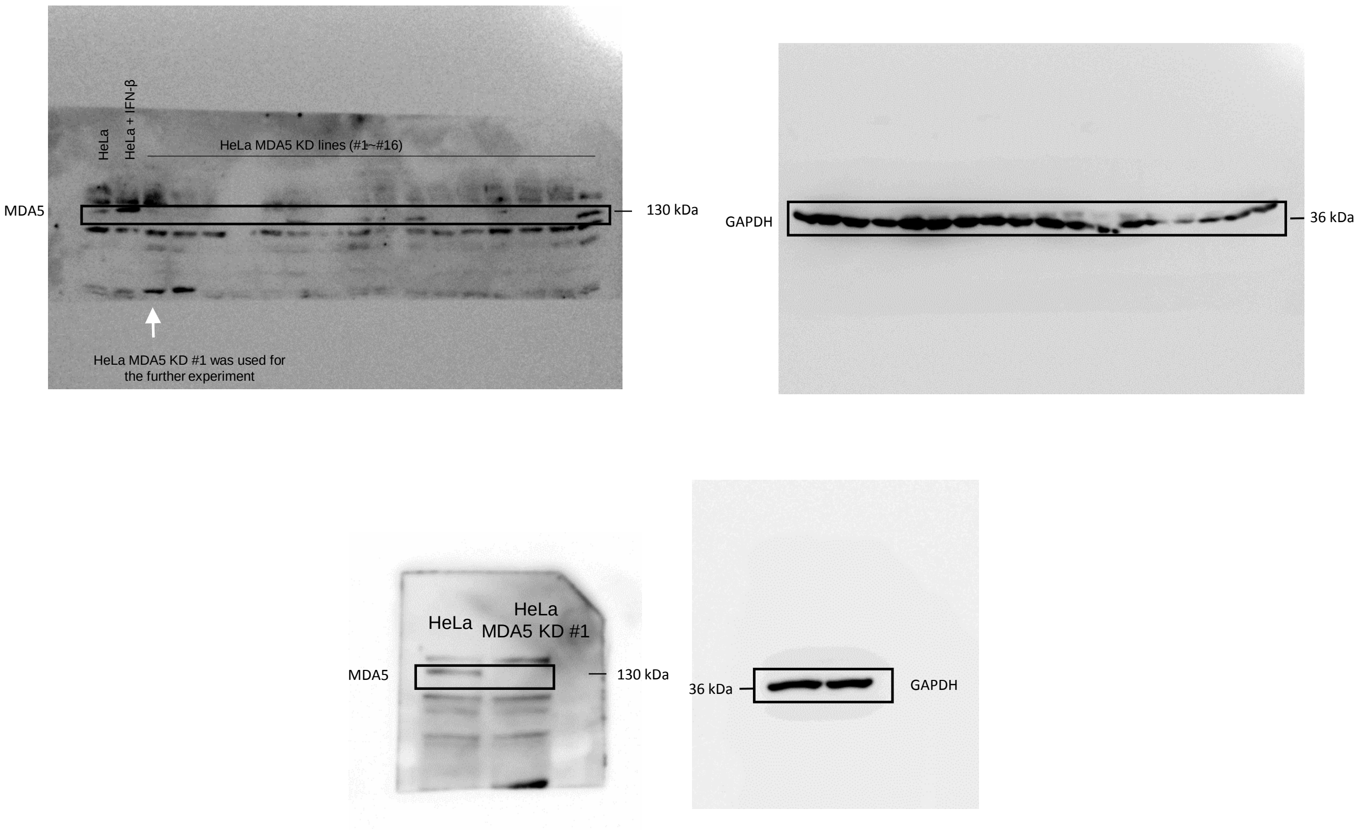

Raw data of Supplementary Fig. S2

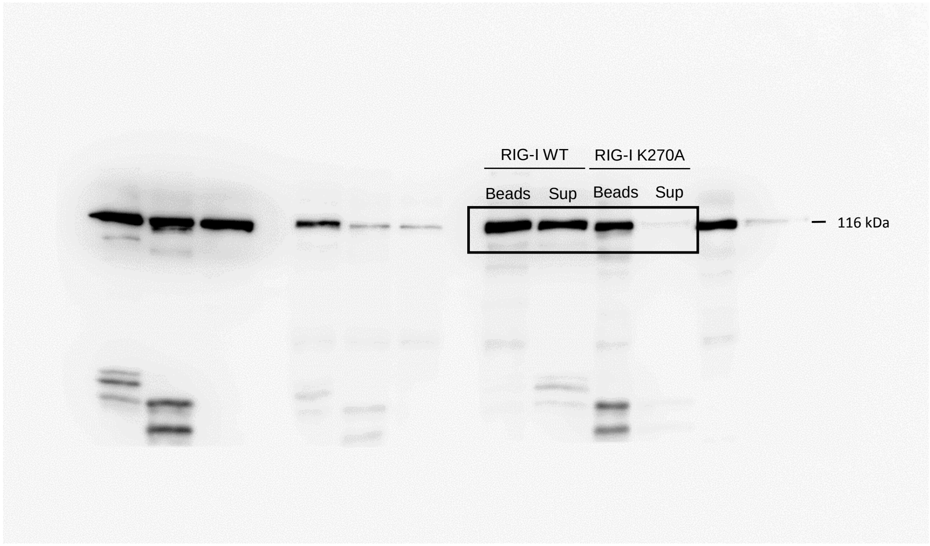

Raw data of Supplementary Fig. S3

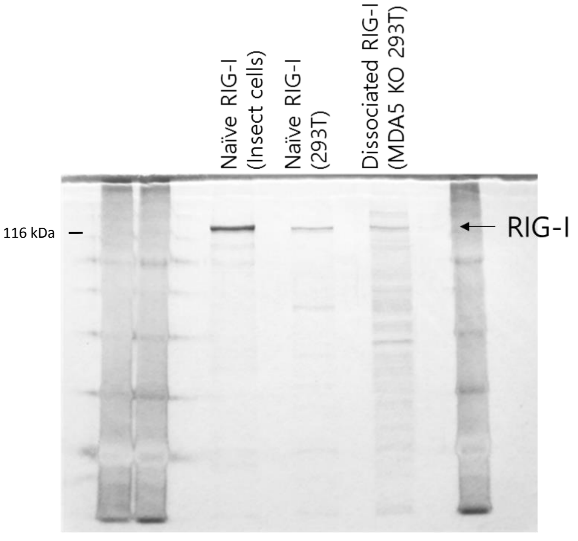

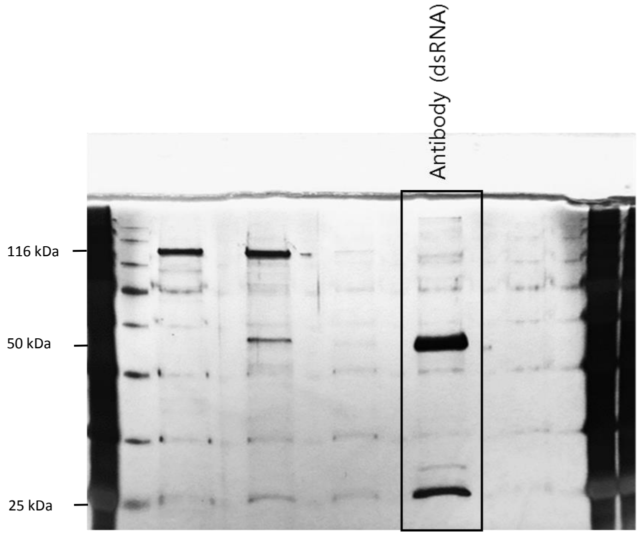

Raw data of Supplementary Fig. S4

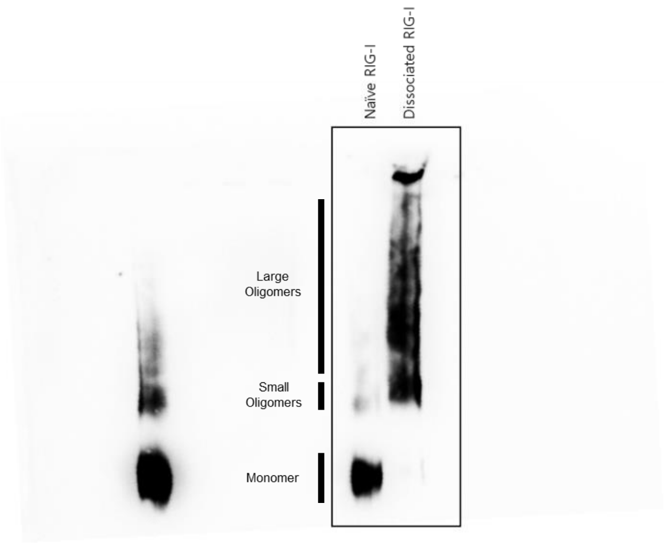

Raw data of Supplementary Fig. S5

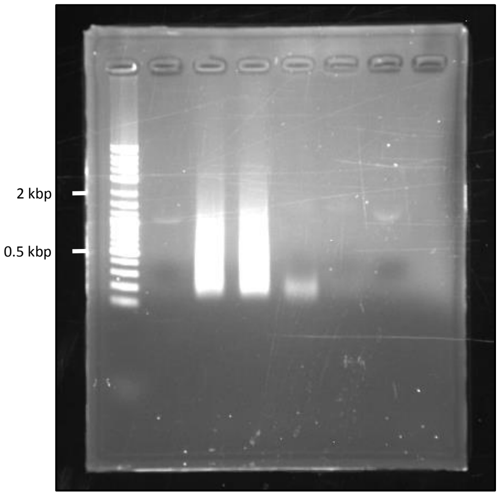

Supplementary Figure S11 shows full-size of an uncropped gel image and a Native-PAGE image in sup plementary Figure 1, 2, 3 and 4 respectively. The membranes were cut as proper size prior to immunoblotting with the antibodies. No more additional modification was done.

Supplementary Table 1

Primer	Sequence (5'→ 3')
human IFNB	Forward: AGCTGCAGCAGTTCCAGAAG<br>Reverse: AGTCTCATTCCAGCCAGTGC
human GAPDH	Forward: CTGCACCACCAACTGCTTAG<br>Reverse: GTCTTCTGGGTGGCAGTGA
